# External validation of prognostic models for chronic kidney disease among type 2 diabetes

**DOI:** 10.1007/s40620-021-01220-w

**Published:** 2022-01-08

**Authors:** Sigit Ari Saputro, Anuchate Pattanateepapon, Oraluck Pattanaprateep, Wichai Aekplakorn, Gareth J. McKay, John Attia, Ammarin Thakkinstian

**Affiliations:** 1 Department of Clinical Epidemiology and Biostatistics, Faculty of Medicine Ramathibodi Hospital, Mahidol University, 270 Rama VI Road, Phayathai, Bangkok, 10400 Thailand; 2 Department of Epidemiology Biostatistics Population and Health Promotion, Faculty of Public Health, Airlangga University, Surabaya, 60115 Indonesia; 3 Department of Community Medicine, Faculty of Medicine Ramathibodi Hospital, Mahidol University, 270 Rama VI Road, Phayathai, Bangkok, 10400 Thailand; 4 Centre for Public Health, School of Medicine, Dentistry and Biomedical Sciences, Queen’s University Belfast, Belfast, UK; 5 School of Medicine and Public Health, and Hunter Medical Research Institute, University of Newcastle, New Lambton, NSW Australia

**Keywords:** External validation, Prognostic model, Chronic kidney disease, Type 2 Diabetes

## Abstract

**Background:**

Various prognostic models have been derived to predict chronic kidney disease (CKD) development in type 2 diabetes (T2D). However, their generalisability and predictive performance in different populations remain largely unvalidated. This study aimed to externally validate several prognostic models of CKD in a T2D Thai cohort.

**Methods:**

A nationwide survey was linked with hospital databases to create a prospective cohort of patients with diabetes (n = 3416). We undertook a systematic review to identify prognostic models and traditional metrics (i.e., discrimination and calibration) to compare model performance for CKD prediction. We updated prognostic models by including additional clinical parameters to optimise model performance in the Thai setting.

**Results:**

Six relevant previously published models were identified. At baseline, C-statistics ranged from 0.585 (0.565–0.605) to 0.786 (0.765–0.806) for CKD and 0.657 (0.610–0.703) to 0.760 (0.705–0.816) for end-stage renal disease (ESRD). All original CKD models showed fair calibration with Observed/Expected (O/E) ratios ranging from 0.999 (0.975–1.024) to 1.009 (0.929–1.090). *Hosmer–Lemeshow* tests indicated a good fit for all models. The addition of routine clinical factors (i.e., glucose level and oral diabetes medications) enhanced model prediction by improved C-statistics of Low’s of 0.114 for CKD and Elley’s of 0.025 for ESRD.

**Conclusions:**

All models showed moderate discrimination and fair calibration. Updating models to include routine clinical factors substantially enhanced their accuracy. Low’s (developed in Singapore) and Elley’s model (developed in New Zealand), outperformed the other models evaluated. These models can assist clinicians to improve the risk-stratification of diabetic patients for CKD and/or ESRD in the regions settings are similar to Thailand.

**Graphical abstract:**

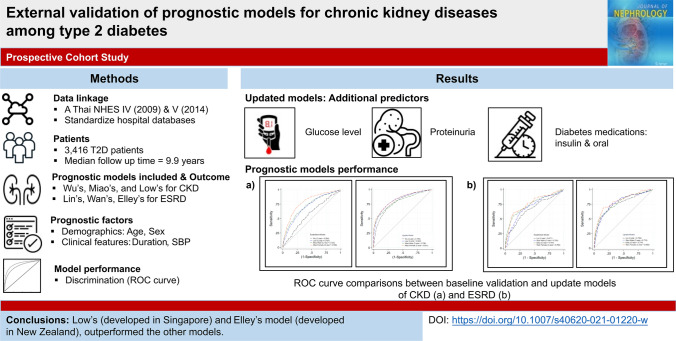

**Supplementary Information:**

The online version contains supplementary material available at 10.1007/s40620-021-01220-w.

## Introduction

Chronic Kidney Disease (CKD) is a major worldwide health burden and the most common microvascular complication of type 2 diabetes (T2D) [1, 2]. In 2017, more than 840 million individuals developed CKD [3], increasing health care demand, particularly in low to middle-income countries (LMICs) [1]. In the UK and the United States, the prevalence of CKD in T2D was reported to range between 25 and 36%, of which 19% was estimated to be advanced (stages 3–5) [4]. The age-standardised global mortality of CKD due to diabetes has been estimated at 7.6 per 100,000[5].

Early detection and treatment are beneficial in the prevention or delay of CKD progression. Despite improved screening, many CKD patients face delayed diagnosis until an advanced stage due to a lack of overt symptoms. Prognostic models for complications associated with T2D progression that incorporate clinical information systems would facilitate improved treatment allocations, healthcare management, and improve understanding of clinical research strategies [6, 7].

Currently, several prognostic equations [8–15] are available for the prediction of CKD in T2D patients, but their generalisability remains uncertain due to limited external and independent validation, particularly in Asian populations [16, 17]. Indeed, external validation is essential and has become mandatory before implementation in clinical practice [16, 18, 19].

Despite many potential advantages, prognostic models have several shortcomings and frequently reported deficiencies [20]. Multiple models have been developed in different ethnicities [8–15, 21–29] but no single model has consistently outperformed all others in Asian populations. For instance, a study based in China performed a limited temporal internal model validation over time on the same data [10]. Most importantly, adaptation of a suitable prognostic model by ethnicity is particularly in an Asian context given that half of the ten countries affected by diabetes worldwide are Asian [4]. Furthermore, recent recommendations have proposed re-evaluation to including race/ethnicity in CKD prediction models [30].

Therefore, this study conducted external validation and improvement of previously published prognostic models of CKD and end stage renal disease (ESRD) in Thai T2D patients.

## Methods

We adhered to the TRIPOD guidelines for the development and validation of a clinical prediction score [31, 32]. We focused on external validation of existing models of CKD-ESRD risk predictions in T2D, supplemented with the addition of routine clinical factors to potentially increase the discriminatory power in our local population [18].

We first identified previous prognostic models by performing a systematic review and meta-analysis (SR/MAs), see Figure S1. We selected prognostic models if they: (1) had been internally or externally validated; (2) reported moderate to excellent discrimination of C-statistics, i.e., ≥ 0.70. We identified six studies that met the inclusion criteria for CKD [8–10] and ESRD [11–13] (Table 1).

**Table 1:** Characteristics of prognostic studies that were used for external validations

Characteristics	Thai NHES	Prognostic score					
		Wu et al. [10]	Miao et al. [9]	Low et al. [8]	Lin et al. [12]	Wan et al. [13]	Elley et al. [11]
Study design	Cross-sectional with updated retrospective data	Prospective cohort	Retrospective cohort	Retrospective cohort	Retrospective cohort	Retrospective cohort	Retrospective cohort
Country	Thailand	China	China	Singapore	Taiwan	Hong Kong	New Zealand
Outcome	CKD-ESRD	CKD	CKD	CKD	ESRD	ESRD	ESRD
Sample size	3416	4795	11,771	1582	24,104	116,509	25,736
Incidence, *n* (%)	CKD:1,383 (43.9) ESRD: 186 (5.9)	590 (12.3)	77 (0.7)	679 (42.9)	1215 (5.04)	238 (0.4)	637 (2.5)
Study settings	Community – hospital-based	Hospital-based	Community – hospital-based	Hospital-based	Hospital-based	Hospital-based	Hospital-based
Study period included	1999 – 2019	2005 – 2010	2014	2002 – 2014	2001 – 2011	2010 – 2015	1988–2010
Model type	Logistic	Logistic	Cox	Logistic	Cox	Cox	Cox
Handling missing data	Excluded	Excluded	Multiple imputation	Excluded	Multiple imputation	MICE	Excluded
Internal validation	n/a	Split sample	Split sample	Split sample	Split sample Bootstrapping	Split sample	
External validation	Yes (standalone external validation)	Yes (temporal external validation)	No	No	No	No	Yes (temporal external validation)
Predicted time, years	n/a	–	5, 10, 20	–	3, 5, 8	5	5
Type 2 diabetes ascertainment	Physical examination at survey (FPG ≥ 126 mg/dL), linked to standard hospital databases (ICD-10) and Laboratory follow up	Medical records, classification based ADA, 2006 (i.e., FPG ≥ 7.0 or OGTT ≥ 11.1 mmol/L)	Medical records, FPG ≥ 7.0 mmol/L, self-report diabetes, or ICD-IX (code 250.X)	Medical records and standardized questionnaire	Medical records, classification based ADA, 2006 (i.e., FPG ≥ 7.0 or OGTT ≥ 11.1 mmol/L)	Medical records based on ICPC version II (code T90)	Medical records, classification based WHO, 1998 (i.e., FPG ≥ 7.0 or OGTT ≥ 11.1 mmol/L)
Outcome ascertainment	ICD-10 code diagnosis, laboratory follow up, death certificate, and physical examination at survey	UACR: 3–30, > 30 (mg/mmol),eGFR-MDRD: < 60 mL/min/1.73m^2^	Persistent albuminuria, medical records review	eGFR-MDRD: < 60 mL/min/1.73 m^2^	ICD-9 (585.x), doubling S-Cr > 2.26 mg/dL, RRT, renal death, or eGFR-MDRD < 15 mL/min/1.73 m^2^	ICD-9 (250.3x, 585.x, 586.x), eGFR < 15 mL/min/1.73 m^2^	Dialysis, RRT, or renal death based on ICD-IX and ICD-10 codes
Number of prognostic factors	n/a	4	8 (Male); 6 (Female)	6	11	10 (Male); 11 (Female)	10 (Equation IV)
Prognostic factors in the final equation	n/a	Sex, BMI, SBP, diabetes duration	Male: age, BMI, Cr, HDL, location, HT/DLP, DR, diet control/physical activity Female: age, Cr, HDL, location, HT/DLP, DR	Age, HbA1c, SBP, UACR, eGFR, LDL-C	Age at initial, Sex, Age on set, Cr, HbA1c, SBP, DR, albuminuria, DM drug, anti-HT drug * SBP/DBP, HLD drug * TC	Male: age, smoking, DR, anti-HT drug, oral DM drug, insulin, HbA1c, SBP, DBP, UACR, eGFR Female: age, duration, anti-HT drug, oral DM drug, insulin, BMI, HbA1c, SBP, DBP, UACR, eGFR	Sex, ethnicity, age on set, duration, Cr, albuminuria, SBP, HbA1c, smoking, CVD history
C-statistic in development phase	n/a	0.713 (0.692 – 0.734)	Male: 0.840 (0.800 – 0.880) Female: 0.800 (0.740 – 0.860)	0.830 (0.790 – 0.870)	0.920 (0.900 – 0.930)	Male: 0.866 (0.849 – 0.882) Female: 0.862 (0.845 – 0.880)	0.914 (0.881 – 0.937)
Calibration	n/a	–	Fair calibration plot	HL-Chi^2^ (*p* value = 0.986)	Fair calibration plot	–	HL-Chi^2^ (*p* value = 0.663)

*ADA* American Diabetes Association, *BMI* body mass index, *CKD* chronic kidney disease, *Cr* creatinine, *CVD* cardiovascular disease, *DBP* diastolic blood pressure, *SBP* systolic blood pressure, *DLP* dyslipidaemia, *DM* diabetic mellitus, *DR* diabetic retinopathy, *eGFR* estimated glomerular filtration rate, *ESRD* end stage renal disease, *FPG* fasting plasma glucose, *HbA1c* glycated haemoglobin, *HDL*-*C* high density lipoprotein cholesterol, *HL* Hosmer-Lemeshow chi square test, *HLD* hyperlipidaemia, *HT* hypertension, *ICD* International classifications of diseases, *ICPC* International classification of primary care, *LDL*-*C* low density lipoprotein cholesterol, *MDRD* Modification of diet in renal disease, *MICE* multiple imputation chained equations, *n*/*a* not appropriate, *NHES* National health examination survey, *OGTT* oral glucose tolerance test, *RRT* renal replacement therapy, *TC* total cholesterol, *UACR* urine albumin creatinine ratio, *WHO* World Health Organization

### Study design and data sources

Data from the Thailand National Health Examination Survey (Thai-NHES) and the standard health databases version 2.4 2019 edition (http://spd.moph.go.th/healthdata/) were used for model validation. The NHES IV and V were population-based cross-sectional surveys conducted in 2009 and 2014, respectively. These surveys captured: health interviews, physical examination, nutrition assessment, and health-related behaviours [33]. Briefly, a multi-stage sampling of adult subjects from the regions, provinces, and districts across the country was used [34, 35].

The standard health databases included medical service records from hospitals, mostly under the direction of the Ministry of Public Health. They comprised a set of tables of all transactions from outpatient and inpatient services for each individual; of 43 files available, only the six that were related to outpatient services (i.e., Person, Diagnosis, Chronic, Drug, Laboratory, and Death) were used for this study.

### Settings and participants

**Fig. 1:**
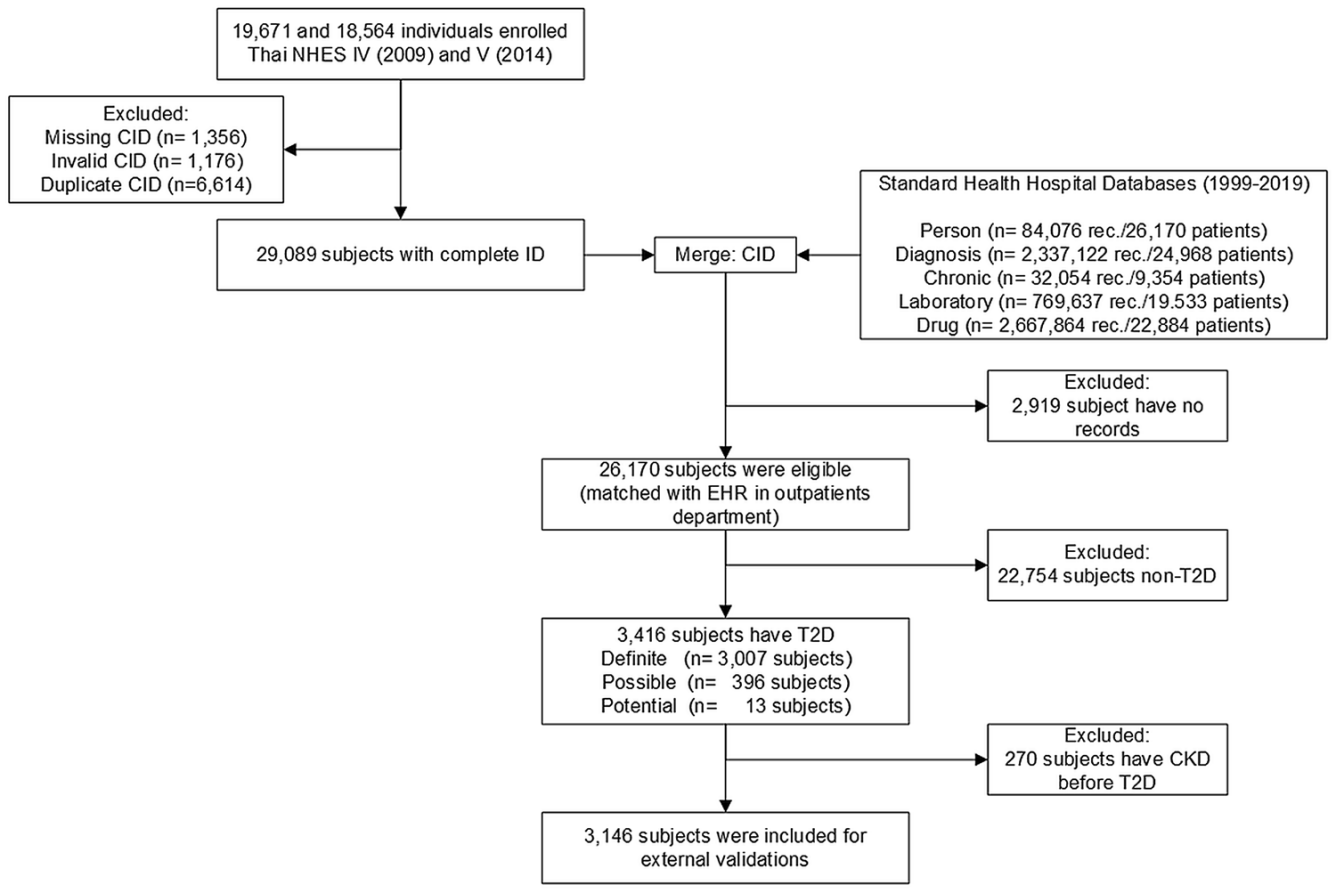
Flowchart for participant inclusion

A total of 19,671 and 18,564 participants were de-identified from NHES IV-V, respectively; removal of duplicates and missing or invalid citizen identification (CID) resulted in 29,089 participants remaining, see Fig. 1. These were linked with the standard hospital health databases (1999–2019) using an encrypted CID to construct the initial sampling frame, leaving a total of 26,170 participants.

We confirmed T2D status based on self-report, medication use, and/or pathology tests (Fasting Plasma Glucose (FPG) ≥ 126 mg/dL or HbA1c ≥ 6.5%). We excluded type 1 diabetes (T1D) with age at onset less than 30 years with severe insulin treatment. There were 3416 participants with identified T2D, of whom 270 (7.9%) were excluded on the basis that CKD was diagnosed prior to T2D, leaving a total of 3146 participants. Of these, 3014 (10.4%) participated in both NHES IV-V, with 402 newly diagnosed participants identified after the survey, see Fig. 1. These participants were followed up from 1999 to October 31st, 2019.

### Outcomes

The primary study outcomes included diabetic nephropathy (CKD stage 3–5) based on the International Classification of Disease, Tenth Edition (ICD-X), which was confirmed by estimated glomerular filtration rate (eGFR) < 60 mL/min/1.73m^2^ measured within 3 months before and after diagnosis, see Table S1. ESRD (CKD stage 5) was defined as eGFR < 15 mL/min/1.73m^2^, or dialysis identified by ICD-X code diagnosis. eGFR was based on the Chronic Kidney Disease Epidemiology Collaboration (CKD-EPI) formula [36].

### Established prognostic factors

We focused on prognostic factors identified through our systematic review, including demographics (age, sex, education, income, and area of residence), biomarkers, comorbidities, medication usage, and clinical features; the latter included diabetes duration, body mass index (BMI; kg/m^2^), waist and hip circumference (cm), systolic/diastolic (SBP/DBP) blood pressure (mmHg), pulse (beat/min), smoking, alcohol consumption, dietary control measures, physical activity, dyslipidaemia, hypertension, and family history of diabetes (FHD, presence of T2D in 1^st^-degree relatives). Biomarkers included lipid profile (i.e., high-density lipoprotein (HDL), low-density lipoprotein (LDL), triglycerides (TG), total cholesterol (TC) in mg/dL, FPG (mg/dL), haemoglobin (g/dL)) and dipstick proteinuria. Comorbidities included a history of cardiovascular disease (CVD) and stroke. CVD was defined by self-report, clinical diagnosis or receipt of treatment for coronary heart disease. Medications recorded included oral-diabetic, blood pressure or cholesterol-lowering drugs.

We included clinical data associated with diabetic complications (i.e., retinopathy, stroke, and composite CVD’s) based on ICD-X diagnostic codes (Table S1), laboratory follow-up, medication treatment (Table S2), or death certification (based on ICD-X).

Hypertension was defined as SBP ≥ 140 or DBP ≥ 90 mmHg or use of anti-hypertensive medication. Dyslipidaemia was defined as HDL ≤ 40 mg/dL, or LDL, TG and TC levels ≥ 130, ≥ 130, and ≥ 200, mg/dL respectively, according to ATP-III guidelines [37].

All factors were included according to their definitions in the original studies (Table S3–S4).

### Statistical analysis

Descriptive statistics for predictor variables were summarised as mean (± standard deviation) or median (interquartile range) for continuous variables or frequency (percentage) for categorical variables. Participant characteristics were compared between groups using Chi-Square or Fisher’s Exact test, where appropriate for categorical variables, and one-way ANOVA or Kruskal Wallis for continuous variables. The predictors which were missing ranged from only 0.1% (n = 3) to 5.8% (n = 199). Therefore, a complete case analysis was applied for the whole analyses.

We evaluated prognostic models originally derived by logistic [8, 10] or Cox regression models [9, 11–13] that were identified from our systematic review (PROSPERO: CRD42018105287). Prognostic scores were calculated according to the published regression formulae using the coefficient and intercept or baseline hazard, see Table S4.

External validation was undertaken in accordance with guidelines for the validation and interpretation of risk prediction models [18, 19]. In brief, we evaluated model performance through comparisons between the original published equation and models that included additional adjustment (e.g., intercept, regression coefficients) for other potential predictors, see Appendix [18, 38–40].

Briefly, model performance was evaluated as follows [7]. Discrimination was assessed by concordance of C-statistics, area under receiver operator characteristic curves (AUROC) [41], and 95% confidence intervals (CI’s). Calibration, i.e., the closeness between the observed and predicted values, was assessed using the *Hosmer–Lemeshow* goodness-of-fit test, the observed to expected (O/E) ratios with 95% CI, and calibration plots. We also used global heuristic shrinkage factors and penalised regression to address issues of over-optimism in updated prognostic models [39, 42, 43].

All statistical analyses were conducted using STATA version 16.0. A two-sided p-value less than 0.05 was considered significant.

## Results

### Characteristics of prognostic models

We identified a total of 6 prognostic studies for CKD-ESRD in T2D patients; see PRISMA flow diagram in Figure S1. Of these, two [8, 10] and four [9, 11–13] applied logistic and Cox regressions, respectively (see Table S4).

Five [8–10, 12, 13] models were developed in Asia and one in New Zealand [11]. Only two [10, 11] models had been externally validated in either a Chinese or New Zealand population. Five [8–13] studies used hospital-based data. The mean age of T2D subjects ranged from 55.4 to 62.9 years with study size ranging between 1582 and 116,509. Five [8–10, 12, 13] studies performed internal validation by splitting samples for discovery and validation, and three [9, 12, 13] applied multiple imputation to account for missing data (see Table 1).

T2D was characterized on the basis of a FPG ≥ 7.0 mmol/L in four [9–12] studies, or medical record review in the remaining two studies [8, 13]. Identification of CKD was mainly based on eGFR and ICD-X codes. The number of prognostic factors included in each model varied between 4 and 11 and included age, sex, SBP, creatinine, and diabetes duration as common predictor variables. These models had fair to good calibration, and discrimination C-statistics ranged between 0.713 [10] and 0.920 [12].

### NHES population characteristics

The T2D cohort included 3,416 participants with a median diabetes duration and follow up time of 5.7 (IQR: 2.6–10.1) and 9.9 (IQR: 6.8–12.7) years, respectively, see Table 1. Of these, 1383 and 186 participants developed CKD and ESRD with an incidence (95% CI) of 43.9% (42.2%, 45.7%) and 5.9% (5.1%, 6.8%), respectively; 704 (22.3%) and 495 (14.5%) developed CVD and retinopathy, and 420 (12.3%) died from any cause.

Baseline characteristics of T2D patients are described in Table 2. The mean (SD) age was 56.6 (12.4) years, and 60.2% were female. The mean age at diabetes onset was 60.0 (12.3) years, and 26.5% of patients had a first degree relative with diabetes. Mean BMI was 26.4 (4.7) kg/m^2^, and the presence of hypertension and dyslipidaemia was 52.3% and 85.2%, respectively.

**Table 2:** Baseline characteristics of T2D in Thailand NHES IV-V

Variables	Missing *n* (%)	All patients (*n* = 3,416)	CKD groups				*P*
			Normal (*n* = 2884)	Stage 3 (*n* = 470)	Stage 4 (*n* = 53)	Stage 5 (*n* = 9)	
Demographic and socio-economic status							
Age, years	–	56.6 (12.4)	54.6 (12.0)	67.8 (8.6)	66.4 (8.0)	64.3 (7.7)	< 0.001^‡^
Age at diabetes onset, years	–	60.0 (12.3)	58.2 (11.9)	70.0 (9.9)	67.4 (9.1)	65.7 (7.4)	< 0.001^‡^
Sex							
Male	–	1360(39.8)	1143(39.6)	189(40.2)	21(39.6)	7(77.8)	0.1^¶^
Female	–	2056(60.2)	1741(60.4)	281(59.8)	32(60.4)	2(22.2)	
Education							
No formal	–	226(6.6)	168(5.8)	55(11.7)	3(5.7)	0(0.0)	< 0.001^§^
Primary	–	2415(70.7)	1999(69.3)	364(77.4)	44(83.0)	8(88.9)	
Secondary	–	543(15.9)	508(17.6)	32(6.8)	3(5.7)	0(0.0)	
University	–	232(6.8)	209(7.2)	19(4.0)	3(5.7)	1(11.1)	
Personal income/month (THB)							
< 5000	–	697(20.4)	644(22.3)	44(9.4)	7(13.2)	2(22.2)	< 0.001^§^
5000 – 10,000	–	545(16.0)	525(18.2)	19(4.0)	1(1.9)	0(0.0)	
10,000 – 25,000	–	450(13.2)	441(15.3)	8(1.7)	1(1.9)	0(0.0)	
≥ 25,000	–	145(4.2)	139(4.8)	4(0.9)	2(3.8)	0(0.0)	
Not answered	–	1579(46.2)	1135(39.4)	395(84.0)	42(79.2)	7(77.8)	
Area of residence							
Rural	–	1608(47.1)	1365(47.3)	213(45.3)	25(47.2)	5(55.6)	0.8^§^
Urban	–	1808(52.9)	1519(52.7)	257(54.7)	28(52.8)	4(44.4)	
Clinical features							
Diabetic duration, years		5.7 (2.6–10.1)	5.5 (2.6–10.1)	7.0 (3.2–13.2)	9.0 (4.8–17.3)	10.4 (4.9–15.3)	< 0.001^‡^
BMI, kg/m^2^	21 (0.6)	26.4 (4.7)	26.6 (4.7)	25.5 (4.3)	25.3 (4.6)	24.3 (7.9)	< 0.001^‡^
Waist circumference, cm	9 (0.2)	87.7 (11.2)	87.8 (11.3)	87.1 (10.7)	87.1 (12.7)	82.4 (14.0)	0.3^†^
Hip circumference, cm	15 (0.4)	97.2 (9.5)	97.5 (9.5)	95.5 (9.2)	95.5 (11.2)	93.4 (15.1)	< 0.001^‡^
Blood pressure							
SBP, mmHg	3 (0.09)	132.6 (19.8)	131.9 (19.3)	136.0 (20.8)	141.1 (29.1)	142.0 (28.6)	< 0.001^‡^
DBP, mmHg	3 (0.09)	78.9 (11.3)	79.4 (11.2)	76.0 (10.8)	79.0 (15.6)	74.8 (10.7)	< 0.001^‡^
Pulse, beat/min	4 (0.12)	78.5 (13.1)	78.5 (12.7)	78.5 (15.1)	77.2 (16.3)	79.2 (8.3)	0.6^‡^
Smoking status							
Non-smoker	–	2375(69.5)	2014(69.8)	324(68.9)	33(62.3)	4(44.4)	< 0.001^¶^
Current smoker	–	495(14.5)	440(15.3)	45(9.6)	8(15.1)	2(22.2)	
Past smoker	–	546(16.0)	430(14.9)	101(21.5)	12(22.6)	3(33.3)	
Alcohol drinking							
No	–	2367(69.3)	1927(66.8)	389(82.8)	44(83.0)	7(77.8)	< 0.001^¶^
Yes	–	1049(30.7)	957(33.2)	81(17.2)	9(17.0)	2(22.2)	
Dietary control							
No	–	2228(65.2)	1885 (65.4)	315 (68.9)	33 (62.3)	6 (66.7)	0.003^¶^
Yes	–	1188(34.8)	999 (34.6)	45 (9.6)	8 (15.1)	3 (33.3)	
Physical activity	25 (0.7)						
Low	–	770(22.7)	615(21.5)	128(27.5)	25(47.2)	2(25.0)	< 0.001^§^
Moderate	–	1208(35.6)	1006(35.1)	179(38.5)	19(35.8)	4(50.0)	
High	–	1413(41.7)	1244(43.4)	158(34.0)	9(17.0)	2(25.0)	
FHD in 1st degree relatives							
No	–	2512(73.5)	2029(70.4)	428(91.1)	47(88.7)	8(88.9)	< 0.001^¶^
Yes	–	904(26.5)	855(29.6)	42(8.9)	6(11.3)	1(11.1)	
Dyslipidaemia							
No	–	505 (14.8)	452 (15.7)	49 (10.4)	3 (5.7)	1 (11.1)	0.006^¶^
Yes	–	2911 (85.2)	2432 (84.3)	421 (89.6)	50 (94.3)	8 (88.9)	
Presence of hypertension							
No	–	1630 (47.7)	1465 (50.8)	149 (31.7)	14 (26.4)	2 (22.2)	< 0.001^§^
Yes	–	1786 (52.3)	1419 (49.2)	321 (68.3)	39 (73.6)	7 (77.8)	
Biomarkers							
FPG, mg/dL	199 (5.8)	138.3 (61.5)	139.1 (61.2)	133.4 (60.8)	140.6 (82.8)	132.5 (60.2)	0.4^‡^
Lipid profile							
HDL-C, mg/dL	80 (2.3)	44.1 (11.2)	44.7 (11.2)	41.3 (10.6)	37.4 (9.2)	40.8 (20.6)	< 0.001^‡^
LDL-C, mg/dL	94 (2.7)	134.1 (41.5)	135.5 (41.3)	126.7 (40.8)	132.9 (52.2)	94.0 (23.6)	< 0.001^‡^
TG, mg/dL	95 (2.8)	158.6 (112.0–227.7)	155.1 (110.8–225.0)	170.6 (119.6–234.8)	181.6 (141.8–244.5)	124.0 (92.0–197.6)	< 0.001^‡^
TC, mg/dL	94 (2.7)	213.2 (47.7)	214.4 (47.7)	206.8 (46.5)	212.0 (55.6)	160.5 (33.8)	< 0.001^†^
Serum creatinine, mg/dL	102 (2.9)	0.9 (0.7–1.0)	0.8 (0.7–1.0)	1.3 (1.1–1.5)	2.2 (2.0–2.7)	5.9 (4.7–11.7)	< 0.001^‡^
eGFR, mL/min/1.73 m^2^	102 (2.9)	84.6 (24.0)	92.2 (17.5)	48.4 (9.0)	24.5 (5.3)	8.2 (4.4)	< 0.001^‡^
Blood Haemoglobin, g/dL	74 (2.1)	13.1 (1.7)	13.3 (1.6)	12.4 (1.7)	11.4 (2.1)	10.7 (1.7)	< 0.001^‡^
Dipstick proteinuria	102 (2.9)						
> Trace	–	2874 (86.7)	2496 (89.0)	346 (76.5)	31 (60.8)	1 (14.3)	< 0.001^§^
> 1 g/dL	–	395 (11.9)	282 (10.1)	94 (20.8)	15 (29.4)	4 (57.1)	
> 3 g/dL	–	45 (1.4)	26 (0.9)	12 (2.7)	5 (9.8)	2 (28.6)	
Drug Usage							
Oral diabetic drug							
No	–	2194(64.2)	1961(68.0)	210(44.7)	19(35.8)	4(44.4)	< 0.001^¶^
Yes	–	1222(35.8)	923(32.0)	260(55.3)	34(64.2)	5(55.6)	
Insulin treatment							
No	–	3136(91.8)	2704(93.8)	394(83.8)	31(58.5)	7(77.8)	< 0.001^§^
Yes	–	280(8.2)	180(6.2)	76(16.2)	22(41.5)	2(22.2)	
Blood-pressure lowering drug							
No	–	2215(64.8)	1983(68.8)	204(43.4)	25(47.2)	3(33.3)	< 0.001^¶^
Yes	–	1201(35.2)	901(31.2)	266(56.6)	28(52.8)	6(66.7)	
Cholesterol-lowering drug							
No	–	2633(77.1)	2271(78.7)	323(68.7)	36(67.9)	3(33.3)	< 0.001^¶^
Yes	–	783(22.9)	613(21.3)	147(31.3)	17(32.1)	6(66.7)	
NSAIDs							
No	–	1223(35.8)	1032(35.8)	167(35.5)	22(41.5)	2(22.2)	0.7^¶^
Yes	–	2193(64.2)	1852(64.2)	303(64.5)	31(58.5)	7(77.8)	
Comorbidities							
Diabetic retinopathy							
No	–	2921 (85.5)	2464 (85.4)	401 (85.3)	47 (88.7)	9 (100.0)	< 0.6^¶^
Yes	–	495 (14.5)	420 (14.6)	69 (14.7)	6 (11.3)	0 (0.0)	
History of CHD							
No	–	3279 (96.0)	2792 (96.8)	430 (91.5)	48 (90.6)	9 (100.0)	< 0.001^§^
Yes	–	137 (4.0)	92 (3.2)	40 (8.5)	5 (9.4)	0 (0.0)	
History of stroke							
No	–	3323 (97.3)	2815 (97.6)	449 (95.5)	51 (96.2)	8 (88.9)	0.02^§^
Yes	–	93 (2.7)	69 (2.4)	21 (4.5)	2 (3.8)	1 (11.1)	

Continuous value is presented as mean (SD) or median (IQ), while categorical was showed as numbers (%) when appropriate

*BMI* body mass index, *CHD* coronary heart diseases, *DBP* diastolic blood pressure, *e*-*GFR* estimated glomerular filtration rate, *FHD* family history of diabetes, *FPG* fasting plasma glucose, *HDL*-*C* high-density lipoprotein cholesterol, *LDL*-*C* low-density lipoprotein cholesterol, *NSAID*s nonsteroidal anti-inflammatory drugs, *SBP* systolic blood pressure, *TC* total cholesterol, *TG* triglycerides *THB* Thai baht rate (₿)

Comparisons (*p* value) were obtained by

^¶^Pearson’s Chi Square

^§^Fisher’s Exact Chi Square Test

^†^One-way ANOVA otherwise

^‡^ANOVA Kruskal Wallis

A total of 1,222 (35.8%), 280 (8.2%), and 1,188 (34.8%) participants were undergoing treatment for diabetes, including oral diabetic medications, insulin, or diet-control, respectively. In general, all prognostic factors including demographics, socioeconomic status, clinical features, biomarkers, treatments, and complications demonstrated significant differences between CKD stages 3–5 (Table 2).

### Participant characteristics comparisons

Participants in our study were slightly younger with fewer males (39.8% vs 43.7%–56.2%) compared to the other six CKD-ESRD studies (Table S5). Mean diabetes duration, BMI, serum creatinine, eGFR and SBP-DBP for our cohort fell within the range reported across the various models but the prevalence of dyslipidemia and hypertension was much higher among our participants. Our cohort had lower FPG and HDL-C, but higher lipid levels (i.e., LDL-C, TG and TC). Moreover, the percentages of anti-hypertensive, anti-hyperlipidaemic and oral diabetic medications were lower than for other reported models.

CKD incidence in our study was similar to that reported by Low and colleagues [8] (i.e., 43.9 vs 42.9%), but much higher than that reported in the remaining studies[9, 10], which ranged from 0.7 to 12.3%. The incidence of ESRD in the study by Lin et al. [12] was comparable to our study (5.04% vs 5.90%), but much higher than the other two studies that reported it [11, 13] (0.4% and 2.5%), see Table 1. The coefficients for the associations between prognostic factors and CKD/ESRD in our cohort were estimated and compared to those in the original models, see Table S6. Our coefficients were mostly similar to the model proposed by Low and colleagues [8], but several predictors (i.e., sex, BMI, location, HDL-C, presence of hypertension, and/or dyslipidemia) were not significant compared to the models proposed by Miao et al. [9]. Most predictors in Wu’s model were also significant in our data; however, the effect sizes were lower for SBP, and diabetes duration and the direction of effect was reversed for BMI. Comparison of the corresponding rank odds ratio of predictors included in their respective CKD models identified creatinine (β = 4.653) and retinopathy (β = 1.045) with the strongest effects for females in Miao’s model, whereas SBP (β = 0.902) and diabetes duration (β = 0.891) were highly associated with CKD in Wu’s models, respectively (Table S6).

For modelling ESRD, only three of the 10 predictors were significant in Elley’s [11] equations, including creatinine, diabetes duration and microalbuminuria, whereas in Wan’s [13] models for female participants, insulin use, oral diabetic drug, and SBP were significantly correlated with ESRD in our multivariate analyses (Table S6).

### External validation

**Fig. 2:**
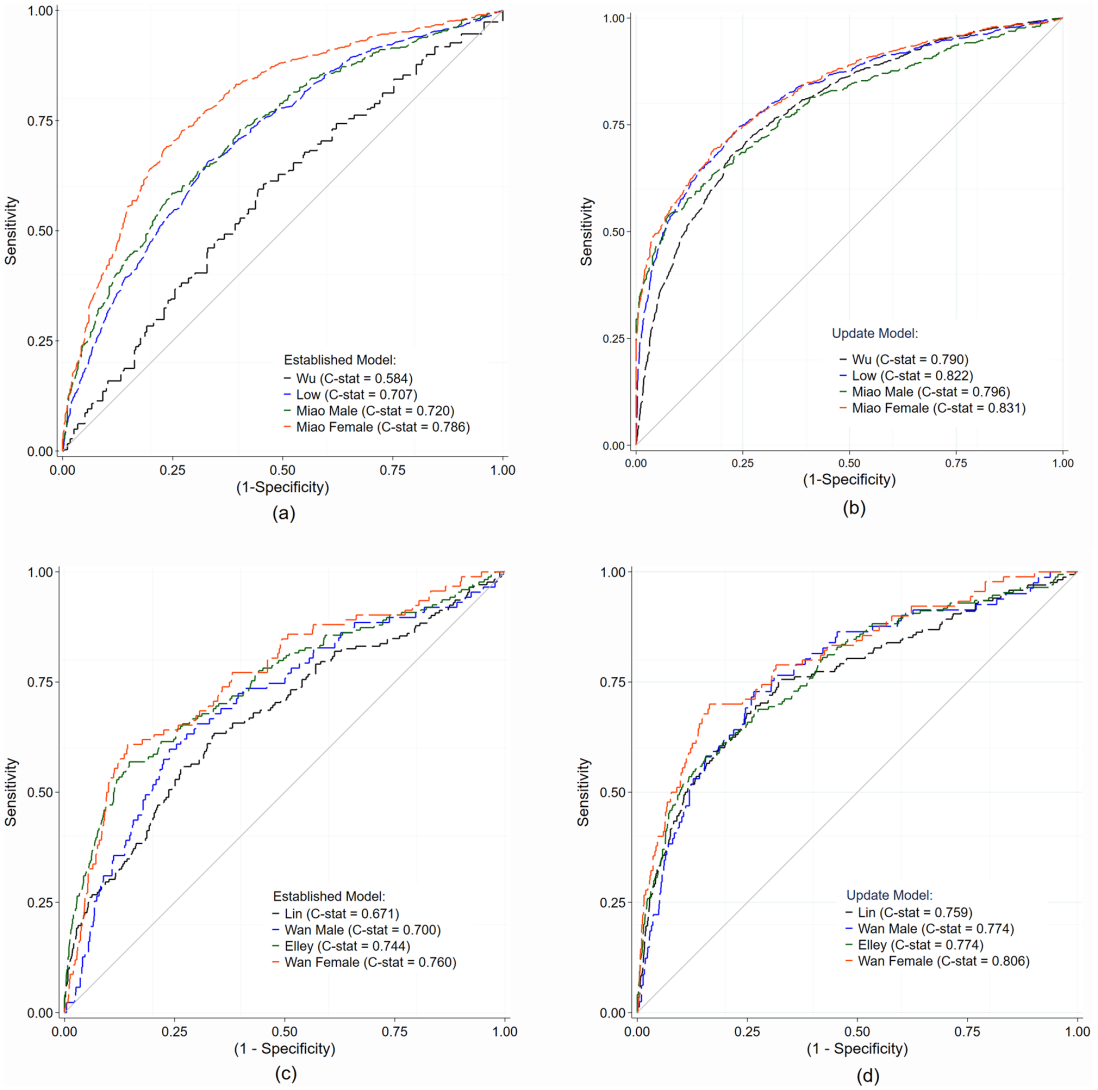
Receiving Operating Characteristic (ROC) curve comparisons between **a** baseline and **b** updated prognostic equations of CKD; and **c** baseline and **d** updated prognostic equations of ESRD

External validations were performed for models M_1_ to M_6_ where applicable (Table S7). Results of CKD-ESRD models are summarised in Table 3. At baseline (M_0_), all prognostic models showed fair calibration, but discrimination varied from poor to moderate, i.e., 0.585 to 0.707 and 0.671 to 0.760 for CKD and ESRD, respectively (Fig. 2). Sex-specific specific CKD and ESRD models performed better for females. For CKD, Miao’s model for females generated a C-statistic of 0.786 (0.765–0.806) compared to 0.720 (0.691–0.749) for males, see Table 3.

**Table 3:** Details of external validation performance for CKD-ESRD models

Study	Derivative	Prognostic factors			External validation								
	C-statistics (95% CI)	n_1_	n_2_	Variables missing/added in the equation	Method	C-statistics (95% CI)	Goodness of fit			O/E ratio (95% CI)	Brier Score	${S}_{VH}$	Penalty (%)
							H–L	df	*P*				
Part A: original prognostic model Chronic kidney disease (CKD)													
Wu et al. [10]	0.713 (0.692–0.734)	4	4	–	M_0_	0.585 (0.565, 0.605)	7.59	8	0.4	0.999 (0.975, 1.024)	0.241	0.985	1.48
			4	–	M_1_	–	–	–	–	–	–	–	–
			4	–	M_2_	0.585 (0.565, 0.605)	7.59	8	0.4	0.999 (0.975, 1.024)	0.241	0.985	1.48
			4	–	M_3_	0.636 (0.616, 0.656)	6.46	8	0.5	1.002 (0.978, 1.026)	0.231	0.994	0.53
			6	FPG groups * oral diabetic drugs^†^	M_4_	0.790 (0.774, 0.806)	16.42	8	0.09	1.003 (0.953, 1.054)	0.185	0.987	1.21
			4	–	M_5_	0.638 (0.618, 0.657)	2.88	8	0.9	1.001 (0.985, 1.017)	0.231	0.994	4.65
			4	–	M_6_	0.632 (0.612, 0.651)	3.94	8	0.7	1.002 (0.985, 1.020)	0.232	0.994	2.70
Miao et al. [9]	0.800 (0.740–0.860) Male equation	8	8	–	M_0_	0.720 (0.691, 0.749)	14.23	8	0.07	1.003 (0.933, 1.074)	0.211	0.994	0.55
			8	–	M_1_	–	–	–	–	–	–	–	–
			8	–	M_2_	0.720 (0.691, 0.749)	14.23	8	0.07	1.003 (0.933, 1.074)	0.211	0.994	0.55
			8	–	M_3_	0.794 (0.768, 0.820)	49.82	8	< 0.001	1.056 (0.859, 1.253)	0.179	0.997	0.27
			10	FPG groups, oral diabetic drugs^†^	M_4_	0.796 (0.769, 0.822)	42.74	8	< 0.001	1.052 (0.868, 1.235)	0.178	0.997	0.28
			8	–	M_5_	0.826 (0.802, 0.850)	28.32	8	< 0.001	1.004 (0.849, 1.159)	0.166	0.982	1.79
			8	–	M_6_	0.824 (0.800, 0.849)	40.80	8	< 0.001	1.125 (0.860, 1.389)	0.165	0.988	1.13
	0.840 (0.800–0.880) Female equation	6	6	–	M_0_	0.786 (0.765, 0.806)	21.55	8	0.006	1.009 (0.929, 1.090)	0.187	0.997	0.21
			6	–	M_1_	–	–	–	–	–	–	–	–
			6	–	M_2_	0.786 (0.765, 0.806)	21.55	8	0.006	1.009 (0.929, 1.090)	0.187	0.997	0.21
			6	–	M_3_	0.816 (0.797, 0.836)	26.05	8	< 0.001	1.040 (0.919, 1.161)	0.169	0.998	0.15
			8	FPG groups * oral Diabetic drugs^†^	M_4_	0.831 (0.812, 0.851)	37.45	8	< 0.001	1.036 (0.917, 1.156)	0.162	0.998	0.15
			6	–	M_5_	0.821 (0.802, 0.841)	34.26	8	< 0.001	1.008 (0.849, 1.167)	0.167	0.990	0.93
			6	–	M_6_	0.820 (0.801, 0.840)	32.19	8	< 0.001	1.004 (0.849, 1.159)	0.167	0.995	0.46
Low et al. [8]	0.830 (0.790–0.870)	6	4	UACR^‡^, HbA1c^‡^	M_0_	0.707 (0.689, 0.726)	14.72	8	0.06	1.000 (0.956, 1.044)	0.216	0.997	0.25
			4	UACR^‡^, HbA1c^‡^	M_1_	0.707 (0.689, 0.726)	14.72	8	0.06	1.000 (0.956, 1.044)	0.216	0.997	0.25
			4	UACR^‡^, HbA1c^‡^	M_2_	0.708 (0.689, 0.730)	14.72	8	0.06	1.000 (0.956, 1.044)	0.216	0.997	0.25
			4	UACR^‡^, HbA1c^‡^	M_3_	0.803 (0.787, 0.819)	69.16	8	< 0.001	1.013 (0.858, 1.169)	0.177	0.999	0.10
			6	FPG groups * Oral Diabetic drugs^†^	M_4_	0.822 (0.806, 0.837)	45.13	8	< 0.001	1.016 (0.875, 1.157)	0.168	0.994	0.53
			4	UACR^‡^, HbA1c^‡^	M_5_	0.810 (0.795, 0.826)	76.69	8	< 0.001	1.010 (0.836, 1.183)	0.172	0.996	0.39
			4	UACR^‡^, HbA1c^‡^	M_6_	0.810 (0.795, 0.826)	76.69	8	< 0.001	1.010 (0.836, 1.183)	0.172	0.996	0.39
End stage renal disease (ESRD)													
Lin et al. [12]	0.920 (0.900–0.930)	11	9	HbA1c‡, SBP (SD) ^‡^	M_0_	0.671 (0.626, 0.717)	17.06	8	0.02	1.026 (0.890, 1.161)	0.052	0.988	1.15
			9	HbA1c^‡^, SBP (SD) ^‡^	M_1_	–	–	–	–	–	–	–	–
			9	HbA1c^‡^, SBP (SD) ^‡^	M_2_	0.671 (0.626, 0.717)	17.06	8	0.02	1.026 (0.890, 1.161)	0.052	0.988	1.15
			9	HbA1c^‡^, SBP (SD) ^‡^	M_3_	0.747 (0.704, 0.790)	15.75	8	0.04	0.986 (0.872, 1.099)	0.051	0.993	0.66
			11	FPG^†^, BMI^†^	M_4_	0.759 (0.716, 0.801)	7.06	8	0.5	0.973 (0.896, 1.051)	0.052	0.993	0.63
			9	HbA1c^‡^, SBP (SD) ^‡^	M_5_	0.774 (0.734, 0.812)	11.12	8	0.2	0.973 (0.863, 1.083)	0.048	0.876	12.31
			9	HbA1c^‡^, SBP (SD) ^‡^	M_6_	0.756 (0.714, 0.798)	5.78	8	0.6	0.976 (0.896, 1.056)	0.049	0.949	5.06
Wan et al. [13]	0.866 (0.849–0.882) Male equation	11	9	UACR^‡^, HbA1c^‡^	M_0_	0.700 (0.639, 0.761)	10.56	8	0.2	1.014 (0.831, 1.197)	0.064	0.975	2.48
			9	UACR^‡^, HbA1c^‡^	M_1_	–	–	–	–	–	–	–	–
			9	UACR^‡^, HbA1c^‡^	M_2_	0.700 (0.639, 0.761)	10.56	8	0.2	1.014 (0.831, 1.197)	0.064	0.975	2.48
			9	UACR^‡^, HbA1c^‡^	M_3_	0.746 (0.690, 0.803)	12.82	8	0.1	0.977 (0.818, 1.135)	0.062	0.984	1.53
			12	Albuminuria^†^, FPG^†^, LDL–C^†^	M_4_	0.774 (0.717, 0.830)	7.40	8	0.4	0.998 (0.838, 1.157)	0.061	0.987	1.28
			9	UACR^‡^, HbA1c^‡^	M_5_	0.758 (0.703, 0.812)	5.77	8	0.6	0.982 (0.869, 1.095)	0.061	0.844	15.55
			9	UACR^‡^, HbA1c^‡^	M_6_	0.747 (0.693, 0.801)	2.22	8	0.5	0.992 (0.913, 1.071)	0.062	0.942	5.71
	0.862 (0.845–0.880) Female equation	11	9	UACR^‡^, HbA1c^‡^	M_0_	0.760 (0.705, 0.816)	12.51	8	0.1	1.042 (0.872, 1.212)	0.045	0.989	1.09
			9	UACR^‡^, HbA1c^‡^	M_1_	–	–	–	–	–	–	–	–
			9	UACR^‡^, HbA1c^‡^	M_2_	0.760 (0.705, 0.816)	12.51	8	0.1	1.042 (0.872, 1.212)	0.045	0.989	1.09
			9	UACR^‡^, HbA1c^‡^	M_3_	0.795 (0.742, 0.847)	2.51	8	0.9	0.981 (0.890, 1.071)	0.042	0.992	0.76
			10	FPG^†^	M_4_	0.806 (0.755, 0.857)	6.65	8	0.5	1.006 (0.865, 1.147)	0.043	0.992	0.73
			9	UACR^‡^, HbA1c^‡^	M_5_	0.820 (0.733, 0.867)	4.49	8	0.8	0.981 (0.868, 1.094)	0.041	0.902	9.70
			9	UACR^‡^, HbA1c^‡^	M_6_	0.789 (0.737, 0.842)	15.26	8	0.05	1.000 (0.895, 1.104)	0.042	0.963	3.61
Elley et al. [11]	0.914 (0.881–0.937)	12	10	UACR^‡^, HbA1c^‡^	M_0_	0.744 (0.701, 0.788)	7.47	8	0.4	0.983 (0.895, 1.070)	0.049	0.994	0.58
			10	UACR^‡^, HbA1c^‡^	M_1_	0.744 (0.701, 0.788)	7.46	8	0.4	0.981 (0.895, 1.068)	0.049	0.994	0.58
			10	UACR^‡^, HbA1c^‡^	M_2_	0.744 (0.701, 0.788)	7.46	8	0.4	0.981 (0.895, 1.068)	0.049	0.994	0.58
			10	UACR^‡^, HbA1c^‡^	M_3_	0.752 (0.709, 0.793)	9.86	8	0.2	0.970 (0.890, 1.051)	0.049	0.994	0.55
			13	BMI^†^, FPG^†^, Oral Diabetic drugs^†^	M_4_	0.774 (0.734, 0.814)	14.25	8	0.07	0.950 (0.838, 1.061)	0.051	0.994	0.53
			10	UACR^‡^, HbA1c^‡^	M_5_	0.762 (0.723, 0.801)	8.76	8	0.3	0.943 (0.812, 1.073)	0.049	0.942	5.73
			10	UACR^‡^, HbA1c^‡^	M_6_	0.761 (0.722, 0.801)	10.05	8	0.2	0.976 (0.896, 1.056)	0.050	0.982	1.70
Part B: simplified prognostic models Chronic kidney disease (CKD)													
Wu et al [10]	0.720 (0.696–0.744)	4	4	–	M_0_	0.585 (0.565, 0.605)	7.72	8	0.4	1.000 (0.976, 1.025)	0.241	0.985	1.47
			4	–	M_1_	–	–	–	–	–	–	–	–
			4	–	M_2_	0.585 (0.565, 0.605)	7.72	8	0.4	1.000 (0.976, 1.025)	0.241	0.985	1.47
			4	–	M_3_	0.603 (0.583, 0.623)	7,58	8	0.4	0.999 (0.975, 1.024)	0.239	0.989	1.00
			6	FPG * oral diabetic drugs^†^	M_4_	0.663 (0.643, 0.682)	8.32	8	0.4	1.000 (0.969, 1.030)	0.227	0.987	1.21
			4	–	M_5_	0.638 (0.619, 0.657)	2.88	8	0.9	1.000 (0.986, 1.014)	0.231	0.953	4.65
			4	–	M_6_	0.633 (0.613, 0.652)	3.94	8	0.8	1.000 (0.971, 1.032)	0.232	0.972	2.70
End Stage renal Disease (ESRD)													
Lin et al. [12]	0.920 (0.900–0.930)	11	9	HbA1c^‡^, SBP (SD)^‡^	M_0_	0.657 (0.610, 0.703)	5.13	8	0.7	1.010 (0.934, 1.086)	0.052	0.987	1.28
			9	HbA1c^‡^, SBP (SD)^‡^	M_1_	–	–	–	–	–	–	–	–
			9	HbA1c^‡^, SBP (SD)^‡^	M_2_	0.657 (0.610, 0.703)	5.13	8	0.7	1.010 (0.934, 1.086)	0.052	0.987	1.28
			9	HbA1c^‡^, SBP (SD)^‡^	M_3_	0.745 (0.703, 0.788)	6.24	8	0.6	0.982 (0.902, 1.062)	0.051	0.993	0.66
			11	FPG^†^, BMI^†^	M_4_	0.756 (0.713, 0.798)	12.99	8	0.1	0.971 (0.860, 1.082)	0.052	0.993	0.63
			9	HbA1c^‡^, SBP (SD)^‡^	M_5_	0.774 (0.734, 0.812)	11.12	8	0.2	0.973 (0.863, 1.083)	0.048	0.876	12.31
			9	HbA1c^‡^, SBP (SD)^‡^	M_6_	0.756 (0.714, 0.798)	5.78	8	0.6	0.976 (0.896, 1.056)	0.049	0.949	5.06

*n*_1_ number of prognostic factors included in derivative, *n*_2_ number of prognostic factors included in external validation, ${S}_{VH}$ Global heuristic shrinkage factor, M1 did not perform if the model did not report the baseline coefficients

^‡^Missing variables

^†^Updated by adding new variables into equation

^*^Interaction effects

All CKD-ESRD models provided improved C-statistics following additional adjustments of the regression coefficient (M_3_) and updated models from (M_4_ – M_6_), see Figure S2. We updated CKD models by adding biomarkers (i.e., FPG groups < 126 vs ≥ 126 mg/dL) and/or interaction effects with oral diabetic drug use; the greatest improvement was observed in the model by Wu and colleagues with a C-statistic of 0.790 (0.774 – 0.806), see Table 3.

In the baseline validation, most CKD models were well-calibrated in our population with O/E ranging from 0.999 (0.975 –1.024) to 1.009 (0.929 – 1.090). Model calibration remained similar after updating, although Miao’s model for males and females showed a slight overestimation of 1.052 (0.868 – 1.235), and 1.036 (0.917 – 1.156), respectively.

Four ESRD risk scores showed moderate to good calibration for baseline validation, recalibration, and updated models, see Figure S3 and Table 3. Fitting the equations using our validation set of ESRD equations (M_5_) showed worsening shrinkage, with a penalty of 12.31% and 15.55% for Lin’s and Wan’s male models, respectively.

The Brier score is another measure of prediction accuracy, ranging between 0 and 1, where lower scores indicate better accuracy. The Brier scores for the baseline and updated models are presented in Table 3. In the updated CKD model, the lowest Brier score was observed in Miao’s model for females (0.162), Low’s model (0.168), Miao’s model for males (0.178), and Wu’s model (0.185). Of the four ESRD models, the Brier score for the updated models (M_4_) was superior and ranged from 0.043 to 0.061.

Table S8 provides a summary of the model improvements implemented following baseline validation. New additional predictor variables (i.e., glucose level and/or interaction with oral diabetic medication) significantly improved the discrimination for the CKD models. The highest improvement was observed in Wu’s models with ∆C-statistic of 0.214 (0.193 – 0.234). Most ESRD models showed minor significant discrimination improvements in the updated models.

## Discussion

We externally evaluated, validated, compared and updated six previously published models for predicting CKD/ESRD in a nationwide cohort of Thai participants with T2D, in line with recent framework guidelines [18, 19, 31, 38]. At baseline, most models provided only modest discrimination of T2D patients who developed CKD/ESRD. Two [10, 12] models demonstrated similar performance to their parent models. All models showed good calibration and upon modification, the agreement between observed and expected risk was fair, with only a few models showing slight overestimation.

In this study, the associations observed between prognostic factors and CKD/ESRD risk in Thai participants with T2D differed from previous studies. For instance, either hypertension or dyslipidaemia, LDL-C, and BMI were negatively associated with CKD risk in some models [8–10], with only a few predictors (i.e., diabetic duration, creatinine, and oral diabetic medications) significantly correlated with ESRD risk. We suspect that the lack of associations or variation in the direction of effect observed between previously reported predictor outcomes may have resulted from heterogeneity among the predictors and outcomes in our data, and that used previously for the development sets. However, we were unable to include two important biomarker predictor variables for four [8, 11–13] models (i.e., UACR and HbA1c) as they were unavailable in our data.

We postulate that the magnitude of the C-statistics and miscalibration observed may be explained by case-mix effects represented by the number of events, predictor effects, and heterogeneity in the population characteristics [19, 44, 45]. Variation of the included predictor variables, and sample size characteristics between derivation and validation settings, are likely responsible for the modest model performance in our population [19, 46].

In general, discrimination and calibration improved in our updated models. Although most models demonstrated lower discrimination in our data compared to their original settings, our updated models showed consistent improvement for all evaluation metrics (i.e., Brier score, shrinkage factor, penalty regression, and C-statistics). Most CKD-ESRD models also showed better reclassification (i.e., ∆C-statistic) for the enhanced models. Despite a lack of existing standards, Pencina et al. proposed that ∆C-statistics greater than 0.01 represents a relevant improvement in model prediction [47, 48]. For our data, all models showed significant improvement on modification, with ∆C-statistics ranging between 0.041 and 0.214 for CKD and 0.025 to 0.089 for ESRD equations.

The Brier score has been proposed as a measure of discrimination and calibration for model validation [49]. In this study, ESRD models performed better compared to those for CKD as determined by Brier scores. Almost every validation and updated model showed improved predictions (as judged by a Benchmark value less than 0.25) [40].

In our updated models, four proved more effective either for the prediction of CKD [8, 9] or ESRD[11, 13] in our population, without the need for recalibration or updated equations. These models consistently exceeded all others in terms of calibration and discrimination, and were more comparable to the derived models. Only Elley’s model [11] provided a web calculator (http://www.nzssd.org.nz/cvd_renal/) to facilitate easier routine clinical practice use.

The strengths of our study include the long-term follow-up of diabetic progression in 26,170 individuals over 20 years, the definition of CKD from multiple data sources, and the evaluation of previously published prognostic models identified from a current SR/MA. This study was based on real world data from a clinical setting that used a broad range of routinely captured potential predictor variables evaluated for prognostic performance of renal outcomes in those with incident diabetes. To our knowledge, this is the first independent validation of CKD-ESRD prognostic models in an Asian population using real world data, beyond the populations from which the models originated. Therefore, our findings should be useful in predicting CKD-ESRD occurrence in other Asian regions where their settings are similar to Thailand.

Our study highlighted that eGFR assessment using creatinine was beneficial to kidney disease surveillance in a Thai population. By avoiding specific race/ethnicity coefficients, our updated models still offered accurate prognostic estimates which could be enhanced further through improved clinical and laboratory standards [30, 50].

Our study has several limitations. Markers of kidney damage, such as albuminuria and cystatin-C were not available in our data and missing data for some predictor variables precluded prognostic risk estimates for some models.

## Conclusions

In conclusion, we have provided an independent external validation of prognostic models for the prediction of incident CKD/ESRD in participants with T2D from Thailand. All evaluated prognostic models showed only moderate discriminative performance, but fair calibration at baseline validation. Updated prognostic scores improved predictive performance in most of the evaluation metrics (i.e., discrimination, calibration, and Brier score). An updated prognostic model for clinical use in Asian populations is provided.

Although no model was excellent, prognostic equations not delimited by sex (i.e., Low’s [8] and Elley’s [11]) performed better in our data and may offer clinical utility as a CKD screening tool in primary care for patients with diabetes.

## Supplementary Information

Below is the link to the electronic supplementary material.

Supplementary file1 (DOCX 34215 KB)
