# Supplementary material for: External validation of prognostic models for chronic kidney disease among type 2 diabetes
Source: J Nephrol. 2022 Jan 8;35(6):1637–53. doi: 10.1007/s40620-021-01220-w (PMC9300508; doi:10.1007/s40620-021-01220-w)

# SUPPLEMENTARY MATERIAL

**Title: External Validation of Prognostic Models for Chronic Kidney Disease among Type 2 Diabetes**

**Authors:**

Sigit Ari Saputro^1,2^, Anuchate Pattanateepapon^1^ Oraluck Pattanaprateep^1^, Wichai Aekplakorn^3^, Gareth J. McKay^4^, John Attia^5^, Ammarin Thakkinstian^1^

^1^Department of Clinical Epidemiology and Biostatistics, Faculty of Medicine Ramathibodi Hospital, Mahidol University, Bangkok, Thailand

^2^Department of Epidemiology Biostatistics Population and Health Promotion, Faculty of Public Health, Airlangga University, Surabaya, Indonesia

^3^Department of Community Medicine, Faculty of Medicine Ramathibodi Hospital, Mahidol University, Bangkok, Thailand

^4^Centre for Public Health, School of Medicine, Dentistry and Biomedical Sciences, Queen’s University Belfast, Belfast, UK

^5^School of Medicine and Public Health, and Hunter Medical Research Institute, University of Newcastle, New Lambton, New South Wales, Australia

**Corresponding author:**

DR. Anuchate Pattanateepapon, D.Eng.

Department of Clinical Epidemiology and Biostatistics,

Faculty of Medicine Ramathibodi Hospital, Mahidol University

270 Rama VI Road, Phayathai,

Bangkok 10400, Thailand.

Email: [anuchate.gab@mahidol.ac.th](mailto:anuchate.gab@mahidol.ac.th)

Telephone: (+66) 0 – 2201 – 0833

**Appendix**

Detailed Methodology of external validation study

**Supplementary Tables**

**Table S1.** National standard healthcare identification codes in Thailand

**Table S2.** Standard medications extracted from the hospital data provided.

**Table S3.**  Summary characteristics for prognostic factor definitions

**Table S4.**  Summary of prognostic model equations considered.

**Table S5.**  Comparisons of baseline characteristics for T2D patients from Thai National Health Examination Survey (NHES) and prognostic model for CKD-ESRD

**Table S6.**  Comparison of intercepts and regression coefficients between development and validation datasets

**Table S7.**  Stepwise intercept and regression coefficients for prognostic model validation (M_0_ – M_6_)

**Table S8.**  Prognostic model discrimination performance comparisons between baseline validation (M_0_) and updated models (M_4_)

**Supplementary Figures**

**Figure S1.** PRISMA flowchart for the identification and selection of CKD prognostic models

**Figure S2.** Stepwise Receiving Operating Characteristic (ROC) Curve for each prognostic model validation (M_0_ – M_6_)

**Figure S3.** Stepwise calibration plots for each prognostic model validation (M_0_ – M_6_)

## Appendix

**Detailed Methodology of external validation study**

First, we set up the predictors for all selected models for validation following the operational definition and equations in the original studies, see Table S3-S4. Afterward, we concurrently compared the magnitude of association (β coefficients) between predictors and the outcome (CKD and or ESRD) using univariate and Multivariate Logistic regression, see Table S6. In addition, we considered the coefficients and odds ratio along with 95% CI for each prognostic model in either before or after recalibration, see Table S6 – S7.

We performed external validation and updated the prognostic models for predicting CKD and ESRD specifically following the proposed conceptual framework as follows as:

M_0_ – Fit the score based on original coefficients or simplified score prognostic model without any adjustment (Model without any re-calibration)

M_1_ – Tuned the intercept (Re-calibrate only the intercept) if the model provided the intercepts or baseline hazard, otherwise skip this step

M_2_ – Fit M_1_ and adjust single factor coefficients for all prognostic factors (Re-calibrate for the overall coefficients)

M_3_ – Fit M_2_ and adjust extra regression coefficients for prognostic factors with different magnitude of associations. Add one or more factors in validation datasets using likelihood ratio test for manual selection.

M_4_ – Fit M_2_ and perform stepwise logistic regression to find the additional prognostic factors that were not included in the original equations. We updated the model using routine biomarkers in clinical practice.

M_5_ – Fit and construct logistic equation by re-estimating all regression coefficients of all predictors based on validation data sets only.

M_6_ – Fit M_5_ and perform stepwise selection to determine one or more prognostic factors that were not included in the derived equations. We constructed this score by adding only significant predictors. This model usually had fewer predictors.

- To update prognostic models, we included the candidate of predictors from the routine clinical features that were available in our primary care settings. Then, we selected most significant predictors and/or with their interactions effect which improved discriminatory ability of the update models. Moreover, we also concerned about clinical utility and interpretable rule-based models to select the most appropriate predictors. We used logistic regression for each step to estimate the score and assess the model performance in terms of discrimination and calibration. We obtained discrimination by evaluating the concordance C-statistic. Classified as no predictive ability, poor, fair, moderate, good, and excellent discrimination if the C statistic was < 0.5, 0.5 to 0.6, 0.6 to 0.7, 0.7 to 0.8, 0.8 to 0.9, > 0.9, respectively[1,2]. Moreover, Delta C-statistics were recognised with 95% confidence interval by bootstrapping of 1,000 replications. Delta C-statistics was suggested greater than 0.01 to determine as meaningful model improvement[3,4]. Calibration was assessed by Hosmer-Lemeshow Chi Square testing for goodness of fit, calibration slope, the agreement between the observed to expected (O/E) ratios, heuristic shrinkage factors ($S_{VH}$) and percent penalisation. We assessed overall performance of the prognostic model by using Brier score. Brier score captured simultaneously discrimination and calibration, with lower score indicating better performance. Brier score showed superiority compared to reclassification for evaluating prediction model in external validation set[5,6].

Brier score is determined as the squared difference between predicted probability and the actual outcome for each observation. It ranges between 0 for perfect model, 0.25 for non-informative model and 1 for no match in prediction and outcome[7,8]. The expected brier score, the mean square prediction error was estimated by[6]:

$Brier Score=\frac{1}{n}\sum_{i=1}^{n} \left( d_{i}-x_{i} \right)^{2}$ (1)

Where $d_{i}$ represent the observed disease outcome of subject $i$ and $x_{i}$ are the predicted probabilities of the diseases corresponding to the $i$ subject.

We evaluated regularisation by shrinkage factor to determine overfitting during validations and update prognostic model. Riley et al., proposed shrinkage factor ≥ 0.9 or penalty regression less than 10% during model development to avoid overestimation[9]. Global heuristic shrinkage $\left( S_{VH} \right)$ for logistic regression was obtained for all predictors’ effects as proposed by Van Houwelingen and Le Cessie, this was defined[10,11]:

$S_{VH}=1-\frac{p}{LR}$ (2)

where p is the total number of predictors for the full set of prognostic factors included in the multivariate model. LR refers to the likelihood ratio by using Chi-Square statistic for fit of the models: $LR=-2\left( lnL_{null}-lnL_{prognostic model} \right)$ in which ${lnL}_{null}$ is the log-likelihood for the model without any prognostic factors (i.e., using intercept only for logit equation), and ${lnL}_{prognostic model}$ is the log-likelihood for the final model.

Another solution of shrinkage, $S$for binary outcome in logistic regression considering the minimum sample size was calculated as follows[9]:

$S=\frac{R_{CS}^{2}}{R_{CS}^{2}+\delta\max\left( R_{CS}^{2} \right)}$ (3)

where $R_{CS}^{2}$ is the prediction model’s maximum possible value or adjusted $R_{Nagelkerke}^{2}$ and $\delta$ is suggested small value of absolute difference ≤0.05; maximum $R_{CS}^{2}$ defined by:

$\max\left( R_{CS}^{2} \right)=1-\exp\left( \frac{2lnLnull}{n} \right)$ (4)

In a logistic equation, ${lnL}_{null}$ is calculated from ${lnL}_{null}=Eln\left( \frac{E}{n} \right)+\left( n-E \right)ln\left( 1-\frac{E}{n} \right)$ where E refers to total number of subjects with the disease outcome and n is the total number of sample size.

In summary, our analysis evaluated and compared prognostic model performances[8,12] for each step of validation with update model from M_0_ to M_6_ by following metrics:

Table A1. Evaluation metrics of prediction models that were applied in validation study

| Evaluation metrics | Measurement | Benchmark | Visualisation | Stata command |
| --- | --- | --- | --- | --- |
| Overall performance | Brier score | Lower value is desirable (ranged from 0 to 1),   - 0: perfect - ≤0.25: non-informative prediction | - | *brier* |
| Discrimination | C-statistic | Higher value is desirable (ranged from 0 to 1)   - ≤0.60: poor - 0.60-0.80: moderate - ≥ 0.80: good/ excellent | ROC curve | *lroc*  *roccomp*  *comproc* |
|  | ∆C-Statistic | Improvement C-statistic from the old into the new model with bootstrapping | - | *comproc* |
| Calibration | Hosmer-Lemeshow | Goodness of fit test for testing consistency between predicted and actual probability using Chi-Square test | - | *estat gof* |
|  | O/E ratio | Higher value is desirable (get closed to 1.0) | Calibration plot | *pmcalplot* |
|  | Global heuristic shrinkage factor $\left( S_{VH} \right)$ | Higher value is desirable (get closed to 1.0)  Riley et al.[9] suggested shrinkage ≥0.9 | - | *pmsampsize* |
|  | Penalised regression | Lower value is desirable  Riley et al.[9] suggested No more than 10% | - | *fitstat* |

**References**

1. Fischer JE, Bachmann LM, Jaeschke R (2003) A readers' guide to the interpretation of diagnostic test properties: clinical example of sepsis. Intensive Care Med 29 (7):1043-1051. doi:10.1007/s00134-003-1761-8

2. Lemeshow H (2000) Assessing the Fit of the Model. In: Applied Logistic Regression. pp 143-202. doi:<https://doi.org/10.1002/0471722146.ch5>

3. Pencina MJ, D'Agostino RB, Pencina KM, Janssens AC, Greenland P (2012) Interpreting incremental value of markers added to risk prediction models. Am J Epidemiol 176 (6):473-481. doi:10.1093/aje/kws207

4. Pencina MJ, D'Agostino RB, Sr., D'Agostino RB, Jr., Vasan RS (2008) Evaluating the added predictive ability of a new marker: from area under the ROC curve to reclassification and beyond. Stat Med 27 (2):157-172; discussion 207-112. doi:10.1002/sim.2929

5. Hilden J, Gerds TA (2014) A note on the evaluation of novel biomarkers: do not rely on integrated discrimination improvement and net reclassification index. Stat Med 33 (19):3405-3414. doi:10.1002/sim.5804

6. Assel M, Sjoberg DD, Vickers AJ (2017) The Brier score does not evaluate the clinical utility of diagnostic tests or prediction models. Diagn Progn Res 1:19. doi:10.1186/s41512-017-0020-3

7. Masconi K, Matsha TE, Erasmus RT, Kengne AP (2015) Independent external validation and comparison of prevalent diabetes risk prediction models in a mixed-ancestry population of South Africa. Diabetol Metab Syndr 7:42. doi:10.1186/s13098-015-0039-y

8. Steyerberg EW, Vickers AJ, Cook NR, Gerds T, Gonen M, Obuchowski N, Pencina MJ, Kattan MW (2010) Assessing the performance of prediction models: a framework for traditional and novel measures. Epidemiology 21 (1):128-138. doi:10.1097/EDE.0b013e3181c30fb2

9. Riley RD, Ensor J, Snell KIE, Harrell FE, Jr., Martin GP, Reitsma JB, Moons KGM, Collins G, van Smeden M (2020) Calculating the sample size required for developing a clinical prediction model. Bmj 368:m441. doi:10.1136/bmj.m441

10. Riley RD, Snell KI, Ensor J, Burke DL, Harrell FE, Jr., Moons KG, Collins GS (2019) Minimum sample size for developing a multivariable prediction model: PART II - binary and time-to-event outcomes. Stat Med 38 (7):1276-1296. doi:10.1002/sim.7992

11. van Smeden M, Moons KG, de Groot JA, Collins GS, Altman DG, Eijkemans MJ, Reitsma JB (2019) Sample size for binary logistic prediction models: Beyond events per variable criteria. Stat Methods Med Res 28 (8):2455-2474. doi:10.1177/0962280218784726

12. Kengne AP, Masconi K, Mbanya VN, Lekoubou A, Echouffo-Tcheugui JB, Matsha TE (2014) Risk predictive modelling for diabetes and cardiovascular disease. Crit Rev Clin Lab Sci 51 (1):1-12. doi:10.3109/10408363.2013.853025

## Supplementary Tables

### **Table S1.** National standard healthcare identification codes in Thailand

| **Diagnosis** | **ICD-10 code** |
| --- | --- |
| Type 2 Diabetes Mellitus (T2D) | E11.0, E11.1, E11.2, E11.3, E11.4, E11.5, E11.6, E11.7, E11.8, E11.9 |
| Chronic Kidney Disease (CKD) | N18.0, N18.3, N18.4, N18.5, N18.9, E11.2, E14.2, N08.3 |
| End-Stage Renal Disease (ESRD) | N18.5 |
| Death due to chronic renal failure | N18.0 – N18.5, N18.9, N19.0 |
| Composite CVD’s | I20 – I25, I46, I50, I51, I52, I60 – I65, I67.2, I68, I69, G45-G46 |
| Diabetic retinopathy | E11.3, E13.3, E14.3, H35, H36.0, H36.8, H28.0 |

### **Table S2.** Standard medications extracted from the hospital data provided.

| **Drug usage in diabetes** | **Items** |
| --- | --- |
| T2D drug treatment | 1. Biguanides (i.e., metformin) 2. Sulfonylureas (i.e., glibenclamide, glipizide, gliclazide, glimepiride) 3. Alpha-glucosidase inhibitors (i.e., voglibose, acarbose, miglitol) 4. Thiazolidinediones (i.e., pioglitazone) 5. Dipeptidyl peptidase/ DPP-4 inhibitors (i.e., sitagliptin, vidagliptin, linagliptin, and gemigliptin) 6. Insulin treatment |
| Blood-pressure lowering drugs | 1. Anti-hypertensive (i.e., doxazosin, methyldopa, prazosin, hydralazine) 2. Diuretics (thiazide, furosemide, amiloride, and spironolactone) 3. Beta-blockers (i.e., propranolol, timolol, metoprolol, atenolol, bisoprolol, and carvedilol) 4. ACE-inhibitors (i.e., captopril, enalapril, lisinopril, perindopril, and quinapril) 5. ARBs (i.e., losartan, valsartan, irbesartan, candesartan, telmisartan, Olmesartan, and azilsartan) |
| Lipid modifying agents | 1. Fibrates 2. Statins (i.e., simvastatin, atorvastatin, rosuvastatin, and pravastatin) 3. Ezetimibe |

### **Table S3.** Summary characteristics for prognostic factor definitions

| **Prognostic Factors** | **Definition of variables that were considered in the models.** |
| --- | --- |
| **Chronic Kidney Disease (CKD)** | |
| Wu, et al. | |
| Sex | Male = 1, Female = 0 |
| BMI | Body mass index (kg/m^2^) at diabetes diagnosis, categorized as: <25.0, 25-27.99, and ≥ 28.0 |
| SBP | The mean of 3-times Systolic blood pressure (mmHg), categorized as: <120, 120-129, 130-139, ≥140 |
| Diabetic Duration | Duration of diabetes in years, categorized as: <5.0, 5.0-9,9, 10-14.9, and ≥15.0 |
| Miao, et al. | |
| Sex | Male = 1, Female = 0 |
| Age | Age at initial examination survey in years divided per 5 years for both male and female |
| BMI | Body mass index (kg/m^2^) |
| Creatinine | Ln (Creatinine) for female and Ln (Creatinine/100) for male |
| HDL-C | HDL-cholesterol at baseline in mmol/L |
| Location | Rural = 1, Urban = 0 |
| HT or DLP | Yes = 1, No = 0  Dyslipidemia: TG ≥ 1.7 mmol/L or LDL-C ≥ 3.2 mmol/L or HDL-C < 0.9 mmol/L in males, or HDL-C < 1.0 mmol/L in females  Hypertension: Average blood pressure between the two measurements ≥ 140/90 mmHg, or a previous diagnosis of hypertension (ICD-IX, 401-405) |
| Retinopathy | Yes = 1, No = 0 |
| Physical activity | Yes = 1, No = 0 |
| Low, et al. | |
| Age | Age at initial examination, per 10 years increase |
| SBP | Systolic blood pressure, per 10 mmHg increase |
| eGFR | Estimated Glomerular Filtration Rate, per 5-ml/min/1.73 m^2^ increase |
| LDL | LDL cholesterol, per 1 mmol/L increase |
| HbA1c | Glycated Hemoglobin A1c, per 1% increase |
| UACR | Log urinary albumin creatinine ratio (per 1 mg/g) |
| **End stage renal disease (ESRD)** | |
| Lin, et al. | |
| Age, years | Age at initial examination survey |
| Sex | Male = 1, Female = 0 |
| Age on set T2D | Age on set of T2D diagnosis categorized as: <45, and ≥45 |
| Creatinine, mg/dL | Urine creatinine in mg/dL categorized as: <2.0, 2.0-4.0 and > 4.0 |
| Variation HbA1c | Variation of HbA1c categorized as: <8.5, 8.5-17.5, >17.5 |
| Variation SBP | Variation on SBP categorized as: <4.4, 4.4-8.7, >8.7 |
| Retinopathy | Yes = 1, No = 0 |
| Albuminuria | Yes = 1, No = 0 |
| Anti-DM medication | No medication, oral only, insulin only, oral agent and insulin |
| Hypertensive medications | Yes = 1, No = 0 with sub-categorized as: SBP<130 and DBP<85, SBP:130-139 or DBP, 85-89, SBP:140-159 or DBP, 90-99, SBP≥160 or DBP≥100 |
| Hyperlipidemia medications | Yes = 1, No = 0 with sub-categorized as: TC<200, 200-239, ≥240 |
| Wan, et al. |  |
| Age, years | Age on diabetes onset (years) |
| Sex | Male = 1, Female = 0 |
| Smoking | Smoker = 1, Non-smoker = 0 |
| Retinopathy | Yes = 1, No = 0 |
| Anti-HT drugs | Yes = 1, No = 0 |
| Insulin used | Yes = 1, No = 0 |
| Oral diabetic drugs | Yes = 1, No = 0 |
| SBP, mmHg | Systolic blood pressure in mmHg |
| DBP, mmHg | Diastolic blood pressure in mmHg |
| Diabetic duration | Diabetic duration in years |
| BMI | Body mass index in kg/m^2^ |
| eGFR | Estimated Glomerular Filtration Rate in ml/min/1.73 m^2^ |
| Elley, et al. | |
| Sex | Female = 1, Male = 0 |
| Ethnicity | Categorized as Maori, Pacific, East-Asian, Indo-Asian, Other’s ethnicity |
| Age of onset T2D | Age on set of T2D diagnosis in years |
| Diabetic duration | Duration of diabetes in years |
| Serum creatinine | Serum creatinine per 10 µmol/L |
| Microalbuminuria | ≥ 2.5 mg/mmol in male or ≥ 3.5 mg/mmol in female |
| Macroalbuminuria | ≥ 30 mg and < 100 mg/mmol |
| Adv. albuminuria | ≥ 100 mg/mmol |
| SBP | Systolic blood pressure per 10 mmHg |
| HbA1c | Glycated hemoglobin per 10 mmol/mol |
| HbA1c, % | Glycated hemoglobin in % |
| Smoking status | Smoking categorized as: Non-smoker, Ex-smoker, current smoker |
| Previous CVD | Yes =1, No = 0 |

### **Table S4.** Summary of prognostic model equations considered.

| **Study, Year** | **Prognostic Equations** | | |
| --- | --- | --- | --- |
| **Chronic Kidney Disease (CKD)** | | | |
| Wu, 2017  (Chinese Diabetic Kidney Disease Risk Score) | $CKD=ln\left( \frac{p}{1-p} \right)=\alpha+0.525 x Male+0.322 x {BMI}_{(25-27.9)}+0.602 x {BMI}_{(\geq28)}+0.626 x {SBP}_{(120-129)}+0.970 x {SBP}_{(130-139)}+1.732 x {SBP}_{(\geq140)}+0.322 x {Duration}_{(5-9.9)}+0.794 x {Duration}_{(10-14.9)}+1.074 x {Duration}_{(\geq15)}$ |  |  |
| Miao, 2017  (Chinese Diabetic Nephropathy Risk Score) | ${CKD}_{Male}=h_{t}=h_{0}\left( t \right)\exp(-0.116x Age-0.094x BMI+2.422 x Creatinine-1.272 x HDL+0.576 x Location+0.688 x HT or DLP+1.386 x Retinopathy+0.615 x physical activity)$ | ${CKD}_{Female}=h_{t}=h_{0}\left( t \right)\exp(-0.162x Age+1.413 x Creatinine-1.309 x HDL-1.049 x Location+0.148 x HT or DLP+1.757 x Retinopathy)$ |  |
| Low, 2017  (Singapore CKD Prediction) | $CKD=ln\left( \frac{p}{1-p} \right)= -6.398+0.182 x Age+0.113 x HbA1c+0.131 SBP+0.524 x\log\left( UACR \right)-0.030 x eGFR+0.270 x LDL$ |  |  |
| **End Stage Renal Disease (ESRD)** | | | |
| Lin, 2017  (Hongkong ESRD Risk Score) | $ESRD=h_{t}=h_{0}\left( t \right)\exp(0.02 x Age-0.01 x Male-0.56 x {Age onset}_{\left( \geq45 \right)}+2.57 x {Creatinine}_{\left( 2.0-4.0 \right)}+2.65 x C{reatinine}_{\left( \geq4.0 \right)}+0.23 {HbA1c}_{\left( 8.5-17.5 \right)}+0.48 x {HbA1c}_{\left( \geq17.5 \right)}+0.24 x S{BP}_{(\geq8.7)}-0.08 x {SBP}_{(4.4-8.7)}+0.88 x retinopathy+0.50 x Albuminuria-0.60 x Oral DM drug+0.45 x Insulin+0.12 x Insulin and Oral DM drug+0.12 x HT {drug}_{\left( No, SBP:130-139, DBP:85-89 \right)}+0.46 x HT {drug}_{\left( No, SBP:140-159, DBP:90-99 \right)}+0.72 x HT {drug}_{\left( No, SBP:\geq160, DBP:\geq100 \right)} +0.99 x HT {drug}_{\left( Yes, SBP<130 \& DBP<85 \right)}+1.06 x HT {drug}_{\left( Yes, SBP:130-139, DBP:85-89 \right)}+1.33 x HT {drug}_{\left( Yes, SBP:140-159, DBP:90-99 \right)}+1.65 x HT {drug}_{\left( Yes, SBP:\geq160, DBP:\geq100 \right)}+0.34 x HLP {drug}_{\left( No, TC:200-239 \right)}+0.53 x HLP {drug}_{\left( No, TC:\geq240 \right)}+0.36 x HLP {drug}_{\left( Yes, TC:<200 \right)}+0.35 x HLP {drug}_{\left( Yes, TC:200-239 \right)}+0.99 x HLP {drug}_{\left( Yes, TC:\geq240 \right)}$ |  |  |
| Wan, 2017  (Chinese ESRD Risk Score) | ${ESRD}_{Male}=h_{t}=h_{0}\left( t \right)\exp(0.058 x Age+0.254 x Smoking+0.385 x retinopathy+0.430 x anti-HT drug+0.314 x Oral DM drug+2.280 x Insulin-0.235 x HbA1c+0.009 x SBP-0.072 x DBP+1.147 x\ln\left( UACR+1 \right)+0.896 x {eGFR}_{\left( 60-89 \right)}+2.170 x {eGFR}_{\left( <60 \right)}-0.030 x \left( Age x Insulin \right)-0.010 x ln(UACR+1))$ | ${ESRD}_{Female}=h_{t}=h_{0}\left( t \right)\exp(0.029 x Age+0.009 x Duration+0.506 x anti-HT drug+0.683 x Oral DM drug+0.548 x Insulin-0.162 x BMI-0.356 x HbA1c+0.009 x SBP-0.833 x DBP+0.371 x\ln\left( UACR+1 \right)+0.693 x {eGFR}_{\left( 60-89 \right)}+4.719 x {eGFR}_{\left( <60 \right)}+0.003 x \left( Age x {eGFR}_{\left( 60-89 \right)} \right)-0.304 x \left( Age x {eGFR}_{\left( <60 \right)} \right))$ |  |
| Elley, 2013  (New Zealand DCS Risk Score) | $ESRD=h_{t}=h_{0}\left( t \right)exp(0.401 x Female+0.821 x Maori+0.296 x Pacific-0.138 x East Asian- 0.004 x Indo Asian-0.223 x Others ethnicity+0.024 x Age on set+0.043 x Duration+0.305 x\frac{Creatinine}{10}+0.644 x Microalbuminuria+1.303 x Macroalbuminuria+2.291 x Advanced Albuminuria+0.049 x\frac{SBP}{10}+0.173 x HbA1c-0.003 x Past smoking+0.252 x Current smoking+0.498 x Previous CVD$ |  |  |

### **Table S5**. Comparisons of baseline characteristics for T2D patients from Thai National Health Examination Survey (NHES) and prognostic model for CKD-ESRD

| **Characteristics** | **Thai NHES** | **Prognostic Score** | | | | | |  |
| --- | --- | --- | --- | --- | --- | --- | --- | --- |
|  |  | **Wu et al.**  **(2017)** | **Miao et. al. (2017)** | **Low et al.**  **(2017)** | **Lin et al. (2017)** | **Wan et al.**  **(2017)** | **Elley et al.**  **(2013)** |  |
| Socio Demographics factors | | | | | | | | |
| Age, years | 56.6 ± 12.4 | 59.4 ± 12.3 | - | 57.3 ± 11.6 | 61.09 ± 10.7 | 62.1 ± 9.9 | 61.0 ± 13.0 |  |
| Age on set, years | 60.0 ± 12.3 | - | 55.4 ± 9.4 | - | 54.4 ± 10.8 | - |  |  |
| Male, % | 1,360 (39.8) | 2,695 (56.2) | 5,068 (43.7) | 916 (57.9) | 11,289 (46.8) | 36,289 (46.7) | 12,752 (49.6) |  |
| Ethnicity | Asian | Asian | Asian | Asian | Asian | Asian | Mixed |  |
| Urban residence | 1,808 (52.9) | - | 4,295 (36.5) | - | - | - | - |  |
| Clinical features | | | | | | | | |
| Diabetic duration, years | 7.6 ± 6.8 | 10.2 ± 7.2 | - | 11.0 ± 9.6 | 6.7± 6.4 | 7.1 ± 6.4 | 4.7 ± 5.9 |  |
| BMI, kg/m^2^ | 26.4 ± 4.7 | 25.1 ± 3.6 | 25.2 ± 3.3 | 26.7 ± 4.9 | 25.7 ± 3.8 | 25.5 ± 4.0 | 31.0 ± 4.9 |  |
| Smoking, % | 1,041 (30.5) | - | 1,546 (27.7) | 608 (38.4) | 3,236 (13.4) | 8,559 (11.0) | 11,203 (43.5) |  |
| SBP, mmHg | 132.6 ± 19.8 | 132.5 ± 16.8 | 146.2 ± 20.1 | 135.9 ± 19.2 | 134.9 ± 17.7 | 134.3 ± 16.2 | 138.0 ± 19.0 |  |
| DBP, mmHg | 78.9 ± 11.3 | 79.8 ± 9.4 | - | - | 80.0 ± 10.5 | 75.2 ± 9.8 | - |  |
| Alcohol consumption | 1,049 (30.7) | - | - | - | 2,186 (9.1) | - | - |  |
| Dietary control | 606 (18.0) | - | 3,471 (29.4) | - | - | - | - |  |
| Physical activity | 2,615 (77.1) | - | 3,471 (29.4) | - | - | - | - |  |
| FHD | 904 (26.5) | 2,595 (54.1) | 2,855 (24.3) | - | - | - | - |  |
| Presence dyslipidemia | 2,911 (85.2) | - | 5,451 (46.3) | - | 6,394 (26.5) | - | - |  |
| Presence hypertension | 1,786 (52.3) | - | 5,454 (46.3) | - | 11,031 (45.7) | 55,719 (71.7) | - |  |
| Biomarkers | | | | | | | | |
| HbA1c, % | - | 8.6 ± 2.0 | - | 8.3 ± 1.9 | 8.2 ± 1.9 | 7.2 ± 1.2 | 7.4 ± 1.6 |  |
| FPG, mg/dL | 138.3 ± 61.5 | 142.3 ± 50.4 | - | - | 171.6 ± 64.8 | - | - |  |
| HDL-C, mg/dL | 44.1 ± 11.2 | - | 57.6 ± 17.0 | - | 46.2 ± 13.9 | - | - |  |
| LDL-C, mg/dL | 134.1 ± 41.5 | - | - | 108.3 ± 34.8 | 117.9 ± 31.2 | 112.1 ± 30.9 | - |  |
| Triglycerides, mg/dL | 190.4 ± 127.5 | 181.8 ± 45.5 | - | 168.3 ± 141.7 | 171.9 ± 130.9 | 141.72 ± 79.7 | - |  |
| Total cholesterol, mg/dL | 213.2 ± 47.7 | 182.1 ± 45.6 | - | - | 195.7 ± 42.0 | - | - |  |
| Creatinine, mg/dL | 0.9 ± 0.6 | - | 0.8 ± 0.3 | - | 1.05 ± 0.5 | - | 0.9 ± 0.2 |  |
| eGFR, mL/min/1.73 m^2^ | 84.6 ± 24.0 | 94.8 ± 24.9 | - | 81.4 ± 34.0 | 73.3 ± 21.7 | - | 77.4 ± 20.0 |  |
| UACR, mg/g | - | 266 ± 822 | - | 76.9 ± 121.7 | - | 60.2 ± 346.9 | - |  |
| Albuminuria | 440 (13.3) | - | - | - | 2,893 (11.9) | - | 9,150 (35.5) |  |
| Drug Usage | | | | | | | | |
| Oral diabetic drug | 1,222 (35.8) | - | 9,107 (77.4) | - | 20,722 (85.9) | 68,171 (87.8) | - |  |
| Insulin usage | 280 (8.2) | - | - | - | 903 (3.7) | 1,912 (2.5) | - |  |
| Blood pressure lowering | 1,201 (35.2) | - | - | 995 (63.2) | 12,544 (52.0) | 59,139 (76.2) | - |  |
| Cholesterol lowering | 783 (22.9) | - | - | - | 9,654 (40.1) | 21,496 (27.7) | - |  |
| Comorbidities | | | | | | | | |
| Retinopathy | 495 (14.5) | - | - | - | 324 (1.3) | 4,072 (5.2) | - |  |
| CVDs | 137 (4.0) | - | 483 (4.1) | - | 2,150 (8.9) | - | 6,392 (24.8) |  |
| Stroke | 93 (2.7) | - | 681 (5.8) | - | 1,176 (4.9) | - | - |  |

Abbreviations: BMI, body mass index; CKD, chronic kidney diseases; CVDs, cardiovascular diseases; DBP, diastolic blood pressure; DLP, dyslipidemia; DM, diabetic mellitus; eGFR, estimated glomerular filtration rate; ESRD, end stage renal disease; FHD, family history of diabetes; FPG, fasting plasma glucose; HDL-C, high density lipoprotein cholesterol; HLD, hyperlipidemia; HT, hypertension; ICD, International Classifications of Diseases; LDL-C, low density lipoprotein cholesterol; MDRD, Modification of Diet in Renal Diseases; MICE, Multiple imputation chained equations; n/a, not appropriate; NHES, National Health Examination Survey; RRT, renal replacement therapy; SBP, systolic blood pressure; UACR, urine albumin creatinine ratio

### **Table S6.** Comparison of Intercepts and Regression coefficients between development and validation datasets

| **Study, Model** | **Prognostic factors** | **Derived dataset** | | | | **External validation dataset** | | | | | |
| --- | --- | --- | --- | --- | --- | --- | --- | --- | --- | --- | --- |
|  |  | **Multivariate** | | | | **Univariate** | | | **Multivariate** | | |
|  |  | **β** | **Exp. β (95%CI)** | ***P*** | **β** | | **Exp. β (95%CI)** | ***P*** | **β** | **Exp. β (95%CI)** | ***P*** |
| **Chronic Kidney Disease (CKD)** | | | | | | | | | | | |
| Wu, Chinese DKD Risk Score | Intercept | - | - | - | - | | - | - | -0.612 | 0.542(0.446-0.657) | <0.001 |
|  | Sex   - Female - Male | Ref  0.525 | Ref  1.690(1.406-2.032) | <0.001 | 0.073 | | 1.075 (0.931-1.241) | 0.3 | 0.001 | 1.001 (0.860-1.163) | 0.9 |
|  | BMI, kg/m^2^   - < 25 - 25 – 28 - ≥ 28 | Ref  0.322  0.602 | Ref  1.380 (1.124-1.695)  1.825 (1.459-2.283) | <0.001  <0.001 | -0.149  -0.535 | | 0.862 (0.724-1.026)  0.586 (0.495-0.694) | 0.09  <0.001 | -0.193  -0.612 | 0.824 (0.688-0.987)  0.541 (0.453-0.648) | 0.03  <0.001 |
|  | SBP, mmHg   - < 120 - 120 – 129 - 130 – 139 - ≥ 140 | Ref  0.626  0.970  1.732 | Ref  1.870 (1.190-2.939)  2.638 (1.696-4.102)  5.561 (3.705-8.617) | <0.001  <0.001  <0.001 | 0.111  0.421  0.801 | | 1.118 (0.910-1.374)  1.524 (1.237-1.877)  2.227 (1.839-2.698) | 0.2  <0.001  <0.001 | 0.191  0.523  0.902 | 1.210 (0.980-1.495)  1.686 (1.358-2.093)  2.464 (2.019-3.008) | 0.07  <0.001  <0.001 |
|  | Diabetic Duration, years   - < 5 - 5 – 9.9 - 10 – 14.9 - ≥ 15 | Ref  0.322  0.794  1.074 | Ref  1.380 (1.014-1.878)  2.211 (1.668-2.932)  2.928 (2.230-3.845) | <0.001  <0.001  <0.001 | 0.226  0.170  0.981 | | 1.254 (1.058-1.487)  1.186 (0.962-1.461)  2.669 (2.115-3.369) | 0.009  0.1  <0.001 | 0.201  0.140  0.891 | 1.223 (1.027-1.456)  1.150 (0.928-1.425)  2.440 (1.920-3.101) | 0.02  0.2  <0.001 |
| Miao, Chinese DN Risk Score (Male Equation) | Intercept | - | - | - | - | | - | - | -1.143 | 0.318 (0.068-1.488) | 0.1 |
|  | Age, years | -0.117 | 0.890 (0.790-0.990) | <0.001 | 0.360 | | 1.434 (1.356-1.516) | <0.001 | 0.240 | 1.272 (1.192-1.356) | <0.001 |
|  | BMI, kg/m^2^ | -0.094 | 0.910 (0.790-0.970) | <0.001 | -0.031 | | 0.968 (0.943-0.994) | 0.01 | -0.018 | 0.982 (0.948-1.016) | 0.2 |
|  | Creatinine, mmol/L | 2.422 | 11.27 (7.670-16.56) | <0.001 | 5.282 | | 196.78(94.99-412.0) | <0.001 | 4.653 | 104.98(47.68-231.1) | <0.001 |
|  | HDL-C, mmol/L | -1.276 | 0.280 (0.170-0.470) | <0.001 | -1.015 | | 0.362 (0.230-0.570) | <0.001 | -1.007 | 0.365 (0.205-0.648) | 0.001 |
|  | Location   - Urban - Rural | Ref  0.576 | Ref  1.780 (1.180-2.690) | <0.001 | -0.221 | | 0.801 (0.638-1.000) | 0.05 | -0.136 | 0.872 (0.657-1.157) | 0.3 |
|  | HT or Dyslipidemia   - No - Yes | Ref  0.688 | Ref  1.990 (1.250-2.730) | <0.001 | 0.810 | | 2.248 (1.469-3.439) | <0.001 | 0.573 | 1.773 (1.006-3.125) | 0.04 |
|  | Retinopathy   - No - Yes | Ref  1.386 | Ref  4.000 (2.740-5860) | <0.001 | 0.739 | | 2.094 (1.492-2.939) | <0.001 | 1.045 | 2.842 (1.860-4.344) | <0.001 |
|  | Diet control/ Physical activity   - No - Yes | Ref  0.615 | Ref  1.850 (1.250-2.730) | <0.001 | -0.362 | | 0.696 (0.531-0.912) | 0.008 | -0.367 | 0.693 (0.496-0.967) | 0.03 |
| Miao, Chinese DN Risk Score (Female Equation) | Intercept | - | - | - | - | | - | - | -19.75 | 0.001 (0.000-0.001) | <0.001 |
|  | Age, years | -0.163 | 0.850 (0.760-0.920) | <0.001 | 0.417 | | 1.517 (1.445-1.594) | <0.001 | 0.327 | 1.388 (1.315-1.465) | <0.001 |
|  | BMI, kg/m^2^ | n/a | n/a | n/a | -0.047 | | 0.953 (0.935-0.972) | <0.001 |  | n/a | n/a |
|  | Creatinine, mmol/L | 1.413 | 4.110 (2.780-6.070) | <0.001 | 4.382 | | 80.02(48.57-131.83) | <0.001 | 3.738 | 42.03(24.74-71.39) | <0.001 |
|  | HDL-C, mmol/L | -1.309 | 0.270 (0.150-0.460) | <0.001 | -0.412 | | 0.662 (0.482-0.909) | 0.01 | -0.053 | 0.947 (0.642-1.399) | 0.7 |
|  | Location   - Urban - Rural | Ref  -1.049 | Ref  0.350 (0.240-0.530) | <0.001 | 0.005 | | 1.005 (0.837-1.206) | 0.9 | 0.195 | 1.216 (0.970-1.524) | 0.08 |
|  | HT or Dyslipidemia   - No - Yes | Ref  0.418 | Ref  1.520 (1.020-2.280) | <0.001 | 0.554 | | 1.741 (1.244-2.436) | 0.001 | -0.198 | 0.819 (0.520-1.291) | 0.4 |
|  | Retinopathy   - No - Yes | Ref  1.758 | Ref  5.800 (3.890-8.940) | <0.001 | 0.485 | | 1.625 (1.256-2.103) | <0.001 | 0.898 | 2.455 (1.789-3.371) | <0.001 |
| Low, Singapore Risk Score | Intercept | -6.398 | - | - | - | | - | - | 2.508 | 12.29 (4.352-34.71) | <0.001 |
|  | Age, years | 0.182 | 1.200 (1.040-1.390) | 0.01 | 0.784 | | 2.191 (2.035-2.357) | <0.001 | 0.181 | 1.198 (1.091-1.316) | <0.001 |
|  | SBP, mmHg | 0.131 | 1.140 (1.050-1.230) | 0.002 | 0.172 | | 1.188 (1.144-1.234) | <0.001 | 0.098 | 1.103 (1.054-1.154) | <0.001 |
|  | eGFR, mL/min/1.73 m^2^ | -0.030 | 0.970 (0.940-0.990) | 0.008 | -0.309 | | 0.734 (0.716-0.752) | <0.001 | -0.273 | 0.760 (0.739-0.783) | <0.001 |
|  | LDL, mmol/L | 0.270 | 1.310 (1.100-1.570) | 0.003 | -0.087 | | 0.915 (0.857-0.978) | <0.001 | -0.108 | 0.897 (0.828-0.971) | 0.008 |
|  | UACR | 0.524 | 1.690 (1.540, 1.850) | <0.001 | - | | - | - | - | - | - |
|  | HbA1c | 0.113 | 1.120 (1.040, 1.210) | 0.004 | - | | - | - | - | - | - |
| **End Stage Renal Disease (ESRD)** | | | | | | | | | | | |
| Lin, Hongkong ESRD Risk Score | Intercept | - | - | - | - | | - | - | -4.809 | 0.008 (0.003-0.020) | <0.001 |
|  | Age, years | 0.020 | 1.020 (n/a) | <0.001 | 0.027 | | 1.027 (1.014-1.040) | <0.001 | 0.032 | 1.033 (1.015-1.051) | <0.001 |
|  | Age on set DM   - < 45 - ≥ 45 | Ref  -0.560 | Ref  0.571 (n/a) | <0.001 | 0.107 | | 1.113 (0.691-1.795) | 0.6 | -0.648 | 0.522 (0.270-1.011) | 0.06 |
|  | Sex   - Female - Male | Ref  -0.010 | Ref  0.990 (n/a) | 0.9 | 0.339 | | 1.404 (1.042-1.893) | 0.02 | 0.171 | 1.187 (0.839-1.678) | 0.3 |
|  | Creatinine, mg/dL   - < 2.0 - 2.0 – 4.0 - > 4.0 | Ref  2.570  2.650 | Ref  13.065 (n/a)  14.154 (n/a) | <0.001  <0.001 | 2.516  - | | 12.38(6.547-23.408)  - | <0.001  - | 1.935  - | 6.930 (3.330-14.422)  - | <0.001  - |
|  | Retinopathy   - No - Yes | Ref  0.880 | Ref  2.410 (n/a) | <0.001 | 0.781 | | 2.183 (1.533-3.107) | 0.001 | 0.703 | 2.020 (1.361-2.998) | <0.001 |
|  | Albuminuria   - No - Yes | Ref  0.500 | Ref  1.648 (n/a) | <0.001 | 1.249 | | 3.487 (2.484-4.895) | <0.001 | 0.812 | 2.253 (1.519-3.342) | <0.001 |
|  | DM medications   - No drug - Oral DM drug - Insulin - Insulin + Oral agent | Ref  -0.600  0.450  0.120 | Ref  0.548 (n/a)  1.568 (n/a)  1.127 (n/a) | 0.004  0.05  0.6 | 0.746  1.955  2.018 | | 2.109 (1.486-2.995)  7.069 (3.812-13.108)  7.526 (4.852-11.674) | <0.001  <0.001  <0.001 | 0.403  1.842  1.507 | 1.496 (0.989-2.265)  6.309 (3.177-12.527)  4.514 (2.689-7.577) | 0.06  <0.001  <0.001 |
|  | Anti-HT medications   - No   SBP<130, DBP<85  SBP:130-139 or DBP:85-89  SBP:140-159 or DBP:90-99  SBP≥160 or DBP≥100   - Yes   SBP<130, DBP<85  SBP:130-139 or DBP:85-89  SBP:140-159 or DBP:90-99  SBP≥160 or DBP≥100 | Ref  0.120  0.460  0.720  0.990  1.060  1.330  1.650 | Ref  1.128 (n/a)  1.584 (n/a)  2.054 (n/a)  2.691 (n/a)  2.886 (n/a)  3.781 (n/a)  5.206 (n/a) | 0.5  0.02  0.005  <0.001  <0.001  <0.001  <0.001 | -0.219  0.206  0.150  -0.257  0.540  0.361  0.810 | | 0.803 (0.466-1.383)  1.229 (0.756-1.999)  1.162 (0.580-2.325)  0.772 (0.417-1.431)  1.716 (0.998-2.950)  1.434 (0.880-2.337)  2.250 (1.286-3.933) | 0.4  0.4  0.6  0.4  0.05  0.1  0.004 | -0.179  0.200  0.097  -0.647  0.011  -0.100  2.397 | 0.835 (0.463-1.508)  1.221 (0.715-2.089)  1.103 (0.500-2.429)  0.523 (0.255-1.073)  1.011 (0.524-1.948)  0.904 (0.512-1.595)  1.270 (0.656-2.469) | 0.5  0.4  0.8  0.07  0.9  0.7  0.4 |
|  | Hyperlipidemia medication   - No   TC: <200 mg/dL  TC: 200-239 mg/dL  TC: > 240 mg/dL   - Yes   TC: <200 mg/dL  TC: 200-239 mg/dL  TC: > 240 mg/dL | Ref  0.340  0.530  0.360  0.350  0.910 | Ref  1.405 (n/a)  1.698 (n/a)  1.433 (n/a)  1.419 (n/a)  2.484 (n/a) | 0.005  0.002  <0.001  0.002  <0.001 | -0.303  -0.664  -0.021  0.347  -0.039 | | 0.737 (0.492-1.107)  0.514 (0.321-0.823)  0.978 (0.608-1.575)  1.415 (0.815-2.457)  0.960 (0.447-2.061) | 0.1  0.006  0.9  0.2  0.9 | -0.062  -0.548  -0.125  0.315  -0.327 | 0.939 (0.606-1.469)  0.577 (0.340-0.981)  0.882 (0.500-1.557)  1.137 (0.715-2.625)  0.720 (0.304-1.706) | 0.7  0.04  0.6  0.3  0.5 |
|  | Variation in HbA1c, %   - < 8.5 - 8.5 – 17.5 - > 17.5 | Ref  0.230  0.480 | Ref  1.258 (n/a)  1.616 (n/a) | 0.01  <0.001 | -  - | | -  - | -  - | -  - | -  - | -  - |
|  | Variation in SBP, %   - < 4.4 - 4.4 – 8.7 - > 8.7 | Ref  -0.080  0.240 | Ref  0.923 (n/a)  1.271 (n/a) | 0.4  0.005 | -  - | | -  - | -  - | -  - | -  - | -  - |
| Wan, Chinese ESRD Risk Score  (Male Equations) | Intercept | - | - | - | - | | - | - | -9.010 | 0.001 (0.000-0.517) | 0.03 |
|  | Age, years | 0.060 | 1.060 (1.050-1.080) | <0.001 | 0.026 | | 1.027 (1.008-1.046) | 0.004 | -0.007 | 0.993 (0.966-1.021) | 0.6 |
|  | Smoking   - Non-Smoker - Smoker | Ref  0.010 | Ref  1.290 (1.110-1.500) | 0.001 | -0.133 | | 0.875 (0.556-1.376) | 0.5 | -0.100 | 0.904 (0.557-1.467) | 0.6 |
|  | Retinopathy   - No - Yes | Ref  0.386 | Ref  1.470 (1.180-1.840) | 0.001 | 0.777 | | 2.176 (1.280-3.697) | 0.004 | 0.653 | 1.922 (1.087-3.399) | 0.02 |
|  | Anti-HT drugs   - No - Yes | Ref  0.433 | Ref  1.540 (1.210-1.970) | 0.001 | 0.489 | | 1.632 (1.043-2.552) | 0.03 | -0.025 | 0.975 (0.580-1.639) | 0.9 |
|  | Insulin used   - No - Yes | Ref  2.281 | Ref  9.790 (1.660-57.62) | 0.01 | 1.365 | | 3.917 (2.264-6.777) | <0.001 | 1.641 | 5.161 (0.279-95.268) | 0.3 |
|  | Oral diabetic drugs   - No - Yes | Ref  0.315 | Ref  1.370 (1.070-1.750) | 0.01 | 0.705 | | 2.024 (1.305-3.138) | 0.002 | 0.161 | 1.175 (0.699-4.972) | 0.5 |
|  | SBP, mmHg | 0.010 | 1.010 (1.010-1.020) | <0.001 | 0.005 | | 1.005 (0.994-1.015) | 0.3 | -0.007 | 0.992 (0.975-1.010) | 0.4 |
|  | DBP, mmHg | -0.074 | 0.930 (0.880-0.980) | 0.009 | 0.005 | | 1.005 (0.987-1.024) | 0.5 | 0.136 | 1.145 (0.947-1.385) | 0.1 |
|  | DBP^2^, mmHg | 0.0003 | 1.0004(1.00-1.0008) | 0.04 | 0.0002 | | 1.000 (0.999-1.001) | 0.6 | -0.0006 | 0.999 (0.998-1.000) | 0.2 |
|  | eGFR, mL/min/1.73 m^2^   - ≥ 90 - 60-89 - < 60 | Ref  0.989  2.169 | Ref  2.450 (2.080-2.880)  8.760 (7.080-10.84) | <0.001  <0.001 | 0.668  2.189 | | 8.934 (4.934-16.177)  8.934 (1.077-3.533) | 0.02  <0.001 | 0.849  2.315 | 2.337 (1.185-4.609)  10.133 (4.521-22.710) | 0.01  <0.001 |
|  | Age*Insulin used | -0.029 | 0.970 (0.950-1.000) | <0.001 | 0.022 | | 1.032 (1.013-1.032) | <0.001 | -0.014 | 0.985 (0.938-1.034) | 0.5 |
|  | HbA1c, % | -0.235 | 0.790 (0.640, 0.980) | 0.04 | - | | - | - | - | - | - |
|  | HbA1c^2^, % | 0.019 | 1.020 (1.010, 1.030) | 0.002 | - | | - | - | - | - | - |
|  | Ln (UACR+1), mg/mmol | 1.147 | 3.150 (2.110, 4.690) | <0.001 | - | | - | - | - | - | - |
| Wan, Chinese ESRD Risk Score  (Female Equations) | Intercept | - | - | - | - | | - | - | -2.945 | 0.052 (0.000-254.5) | 0.5 |
|  | Age, years | 0.034 | 1.030 (1.020-1.050) | <0.001 | 0.028 | | 1.028 (1.010-1.046) | 0.002 | -0.022 | 0.977 (0.934-1.022) | 0.3 |
|  | Diabetic durations, years | 0.011 | 1.010 (1.000-1.020) | 0.04 | 0.050 | | 1.051 (1.028-1.074) | <0.001 | -0.027 | 0.973 (0.938-1.000) | 0.1 |
|  | Anti-HT drugs   - No - Yes | Ref  0.503 | Ref  1.660 (1.210-2.260) | 0.002 | 0.235 | | 1.265 (0.834-1.917) | 0.2 | -0.197 | 0.820 (0.487-1.381) | 0.4 |
|  | Insulin used   - No - Yes | Ref  0.545 | Ref  1.730 (1.300-2.290) | <0.001 | 1.943 | | 6.979(4.394-11.085) | <0.001 | 1.658 | 5.250 (3.003-9.179) | <0.001 |
|  | Oral diabetic drugs   - No - Yes | Ref  0.648 | Ref  1.980 (1.430-2.750) | <0.001 | 1.072 | | 2.921 (1.909-4.470) | <0.001 | 0.664 | 1.942 (1.090-3.460) | 0.02 |
|  | BMI, kg/m^2^ | -0.015 | 0.850 (0.770-0.950) | 0.002 | -0.062 | | 0.939 (0.896-0.984) | 0.008 | -0.016 | 0.983 (0.664-1.456) | 0.9 |
|  | BMI^2^, kg/m^2^ | 0.003 | 1.003 (1.001-1.005) | 0.001 | -0.001 | | 0.998 (0.997-0.999) | 0.01 | -0.0007 | 0.999 (0.992-1.006) | 0.8 |
|  | SBP, mmHg | 0.005 | 1.010 (1.000-1.010) | 0.02 | 0.014 | | 1.014 (1.004-1.023) | 0.004 | 0.019 | 1.019 (1.003-1.035) | 0.01 |
|  | DBP, mmHg | -0.086 | 0.920 (0.860-0.980) | 0.007 | -0.009 | | 0.990 (0.971-1.010) | 0.3 | -0.018 | 0.982 (0.820-1.176) | 0.8 |
|  | DBP^2^, mmHg | 0.0005 | 1.001 (1.000-1.001) | 0.01 | -0.00006 | | 0.999 (0.999-1.001) | 0.4 | <0.001 | 1.000 (0.998-1.001) | 1.0 |
|  | eGFR, mL/min/1.73 m^2^   - ≥ 90 - 60-89 - < 60 | Ref  0.693  4.719 | Ref  2.000(0.380-10.51)  112.1(23.97-523.76) | 0.4  <0.001 | 0.415  2.319 | | 1.514 (0.836-2.741)  10.168(6.009-17.207) | 0.1  <0.001 | 0.818  3.805 | 2.268 (0.083-61.251)  44.922 (1.346-1499.2) | 0.6  0.03 |
|  | Age interaction term:  Age* eGFR (60-89)  Age* eGFR (<60) | 0.003  -0.031 | 1.003 (0.979-1.028)  0.970 (0.950-0.990) | 0.8  0.006 | -0.009  0.029 | | 0.990 (0.982-0.998)  1.029 (1.023-1.036) | 0.01  <0.001 | -0.004  -0.020 | 0.996 (0.938-1.057)  0.979 (0.922-1.040) | 0.9  0.5 |
|  | HbA1c, % | -0.356 | 0.700 (0.490, 0.990) | 0.04 | - | | - | - | - | - | - |
|  | HbA1c^2^, % | 0.029 | 1.030 (1.010, 1.050) | 0.01 | - | | - | - | - | - | - |
|  | Ln (UACR+1), mg/mmol | 0.371 | 1.450 (1.370, 1.530) | <0.001 | - | | - | - | - | - | - |
| Elley, New Zealand DCS Risk Score | Intercept | - | - | - | - | | - | - | -4.202 | 0.014 (0.001-0.193) | 0.001 |
|  | Age DM onset, years | 0.024 | 1.020 (n/a) | <0.001 | 0.015 | | 1.016 (1.003-1.028) | 0.01 | -0007 | 0.992 (0.977-1.008) | 0.3 |
|  | Sex   - Female - Male | 0.401  Ref | 1.490 (n/a)  Ref | <0.001 | -0.319 | | 0.727 (0.540-0.978) | 0.03 | 0.170 | 1.185 (0.769-1.825) | 0.4 |
|  | Ethnicity   - European - Maori - Pacific - East Asian - Indo Asian - Others’ ethnicity | Ref  0.821  0.296  -0.138  -0.040  -0.223 | Ref  2.270 (n/a)  1.340 (n/a)  0.870 (n/a)  0.960 (n/a)  0.800 (n/a) | 0.001  <0.001  >0.05  >0.05  >0.05 | -  -  -0.824  -  - | | -  -  0.439 (0.054-3.584)  -  - | -  -  0.4  -  - | -0.921 | 0.398 (0.045-3.505) | 0.4 |
|  | Diabetic duration, years | 0.043 | 1.040 (n/a) | <0.001 | 0.044 | | 1.045 (1.028-1.062) | <0.001 | 0.029 | 1.030 (1.010-1.049) | 0.002 |
|  | Serum Creatinine, µmol/L | 0.305 | 1.360 (n/a) | <0.001 | 0.210 | | 1.234 (1.188-1.281) | <0.001 | 0.203 | 1.225 (1.172-1.278) | <0.001 |
|  | Albuminuria   - Normo-albuminuria - Microalbuminuria - Macroalbuminuria - Adv. Albuminuria | Ref  0.644  1.303  2.290 | Ref  1.900 (n/a)  3.680 (n/a)  9.880 (n/a) | <0.001  <0.001  <0.001 | 1.190  1.758  - | | 3.289 (2.30-4.690)  5.804(2.598-12.965)  - | <0.001  <0.001 | 0.646  0.815 | 1.909 (1.274-2.860)  2.261 (0.827-6.179) | 0.002  0.1 |
|  | SBP, mmHg | 0.049 | 1.050 (n/a) | <0.001 | 0.100 | | 1.105 (1.031-1.185) | 0.005 | 0.029 | 1.029 (0.948-1.117) | 0.4 |
|  | Smoking   - Nonsmoker - Ex Smoking - Current smoking | Ref  -0.003  0.252 | Ref  1.290 (n/a)  1.000 (n/a) | <0.001  <0.001 | 0.276  0.063 | | 1.318 (0.897-1.938)  1.065 (0.694-1.637) | 0.2  0.7 | -0.016  -0.125 | 0.983 (0.592-1.630)  0.883 (0.501-1.553) | 0.9  0.6 |
|  | Previous CVD history   - No - Yes | Ref  0.498 | Ref  1.650 (n/a) | <0.001 | 0.380 | | 1.462 (1.053-2.030) | 0.02 | 0.185 | 1.203 (0.828-1.749) | 0.3 |
|  | HbA1c, %  HbA1c, mmol/mol per 10 | 0.173  0.158 | 1.189 (n/a)  1.171 (n/a) | <0.001  <0.001 | -  - | | -  - | -  - | -  - | -  - | -  - |

Abbreviations: BMI, body mass index; CVD, cardiovascular disease; DBP, diastolic blood pressure; DLP, dyslipidemia; DM, diabetic mellitus; eGFR, estimated glomerular filtration rate; HbA1c, glycated hemoglobin; HDL-C, high density lipoprotein- cholesterol; HT, hypertension; LDL, low density lipoprotein; SBP, systolic blood pressure; TC, Total Cholesterol; UACR, urine albumin to creatinine ratio

### **Table S7.** Stepwise intercept and regression coefficients for prognostic model validation (M_0_ – M_6_)

| **Prognostic Models** | **Prognostic Factors** | **Updating Methods** | | | | | | |
| --- | --- | --- | --- | --- | --- | --- | --- | --- |
|  |  | **M0** | **M1** | **M2** | **M3** | **M4** | **M5** | **M6** |
| **Chronic Kidney Diseases (CKD)** | | | | | | | | |
| Wu, Chinese DKD Risk Score | Intercept | - | - | -0.835 | -0.835 | -0.835 | -0.612 | -0.434 |
|  | Sex   - Female - Male | Ref  0.525 | -  - | Ref  0.180 | Ref  0.180 | Ref  0.180 | Ref  0.0004 | -  - |
|  | BMI, kg/m^2^   - < 25 - 25 – 28 - ≥ 28 | Ref  0.322  0.602 | -  -  - | Ref  0.110  0.206 | Ref  -0.234  -0.679 | Ref  0.110  -0.027 | Ref  -0.193  -0.613 | -  -0.188  -0.600 |
|  | SBP, mmHg   - < 120 - 120 – 129 - 130 – 139 - ≥ 140 | Ref  0.626  0.970  1.732 | -  -  -  - | Ref  0.214  0.333  0.594 | Ref  0.249  0.611  1.169 | Ref  0.214  0.580  1.070 | Ref  0.191  0.522  1.902 | Ref  -  0.438  0.815 |
|  | Diabetic Duration, years   - < 5 - 5 – 9.9 - 10 – 14.9 - ≥ 15 | Ref.  0.322  0.794  1.074 | -  -  -  - | Ref  0.110  0.272  0.368 | Ref  0.236  0.213  1.051 | Ref  -0.590  -1.306  -0.809 | Ref  0.201  0.140  0.892 | Ref  -  -  0.797 |
|  | Age, years  Oral diabetic drug  FPG group ≥ 126 mg/dL  Oral DM drug*FPG group | -  -  -  - | -  -  -  - | -  -  -  - | -  -  -  - | 0.072  2.195  0.971  -0.925 | -  -  -  - | -  -  -  - |
| Miao, Chinese DN Risk Score (Male Equation) | Intercept | - | - | 0.003 | 0.003 | 0.003 | 0.318 | 0.293 |
|  | Age, years | 0.890 | - | 1.033 | 2.320 | 2.278 | 1.272 | 1.286 |
|  | BMI, kg/m^2^ | 0.910 | - | 1.056 | 1.056 | 1.056 | 0.981 | - |
|  | Creatinine, mmol/L | 11.27 | - | 13.079 | 118.157 | 152.751 | 104.978 | 105.078 |
|  | HDL-C, mmol/L | 0.280 | - | 0.324 | 0.670 | 0.769 | 0.365 | 0.346 |
|  | Location   - Urban - Rural | Ref  1.780 | -  - | Ref  2.065 | Ref  2.065 | Ref  2.065 | Ref  0.872 | -  - |
|  | HT or Dyslipidemia   - No - Yes | Res  1.990 | -  - | Ref  2.309 | Ref  2.309 | Ref  4.316 | Ref  1.773 | -  - |
|  | Retinopathy   - No - Yes | Ref  4.000 | -  - | Ref  4.642 | Ref  7.503 | Ref  7.028 | Ref  2.842 | Ref  2.860 |
|  | Diet control/ Physical activity   - No - Yes | Ref  1.850 | -  - | Ref  2.147 | Ref  2.851 | Ref  2.845 | Ref  0.692 | Ref  0.705 |
|  | FPG groups (≥126 mg/dL)  Oral diabetic drug | -  - | -  - | -  - | -  - | 2.021  2.071 | -  - | -  - |
| Miao, Chinese DN Risk Score (Female Equation) | Intercept | - | - | 0.00002 | 0.00002 | 0.00002 | 0.00002 | 0.00002 |
|  | Age, years | 0.850 | - | 1.214 | 2.591 | 2.572 | 1.388 | 1.377 |
|  | Creatinine, mmol/L | 4.110 | - | 5.871 | 47.759 | 52.198 | 42.032 | 41.888 |
|  | HDL-C, mmol/L | 0.270 | - | 0.385 | 0.385 | 0.385 | 0.947 | - |
|  | Location   - Urban - Rural | Ref  0.350 | -  - | Ref  0.499 | Ref  0.499 | Ref  1.787 | Ref  1.216 | -  - |
|  | HT or Dyslipidemia   - No - Yes | Ref  1.520 | -  - | Ref  2.171 | Ref  2.171 | Ref  2.171 | Ref  0.819 | -  - |
|  | Retinopathy   - No - Yes | Ref  5.80 | -  - | Ref  8.285 | Ref  10.691 | Ref  10.124 | Ref  2.455 | -  2.406 |
|  | FPG groups (≥126 mg/dL)  Oral diabetic drugs  Oral DM drug*FPG group | -  -  - | -  -  - | -  -  - | -  -  - | 1.973  3.830  0.534 | -  -  - | -  -  - |
| Low, Singapore Risk Score | Intercept | -6.398 | -1.974 | -1.974 | -1.974 | -1.974 | 2.508 | 2.508 |
|  | Age, years | 0.182 | 0.182 | 0.256 | 0.437 | 0.390 | 0.181 | 0.181 |
|  | SBP, mmHg | 0.131 | 0.131 | 0.184 | 0.282 | 0.284 | 0.098 | 0.098 |
|  | eGFR, mL/min/1.73 m^2^ | -0.034 | -0.034 | -0.042 | -0.315 | -0.323 | -0.273 | -0.273 |
|  | LDL, mmol/L | 0.273 | 0.273 | 0.383 | 0.274 | 0.383 | -0.108 | -0.108 |
|  | UACR | 0.524 | - | - | - | - | - | - |
|  | HbA1c | 0.113 | - | - | - | - | - | - |
|  | FPG groups (≥126 mg/dL)  Oral diabetic drugs  Oral DM drug*FPG group | -  -  - | -  -  - | -  -  - | -  -  - | 0.731  1.137  -0.535 | -  -  - | -  -  - |
| **End Stage Renal disease (ESRD)** | | | | | | | | |
| Lin, Hongkong ESRD Risk Score | Intercept | - | - | -4.084 | -4.084 | -4.084 | -4.809 | -4.768 |
|  | Age, years | 0.020 | - | 0.015 | 0.048 | 0.038 | 0.033 | 0.035 |
|  | Age on set DM   - < 45 - ≥ 45 | Ref  -0.560 | -  - | Ref  -0.410 | Ref  -1.104 | Ref  -0.410 | Ref  -0.648 | Ref  -0.703 |
|  | Sex   - Female - Male | Ref  -0.010 | -  - | Ref  -0.007 | Ref  -0.007 | Ref  -0.007 | Ref  0.171 | -  - |
|  | Creatinine, mg/dL   - < 2.0 - 2.0 – 4.0 - > 4.0 | Ref  2.570  2.650 | -  -  - | Ref  1.882  1.940 | Ref  3.141  1.940 | Ref  3.800  1.940 | Ref  1.935  - | Ref  1.994  - |
|  | Retinopathy   - No - Yes | Ref  0.880 | -  - | Ref  0.644 | Ref  1.360 | Ref  1.274 | Ref  0.703 | Ref  0.764 |
|  | Albuminuria   - No - Yes | Ref  0.500 | -  - | Ref  0.366 | Ref  1.164 | Ref  1.051 | Ref  0.812 | Ref  0.865 |
|  | DM medications   - No drug - Oral DM drug - Insulin - Insulin + Oral agent | Ref  -0.600  0.450  0.120 | -  -  -  - | Ref  -0.439  0.329  0.087 | Ref  -0.028  2.198  1.623 | Ref  -0.016  0.751  1.982 | Ref  0.403  1.842  1.507 | Ref  -  1.715  1.280 |
|  | Anti-HT medications   - No   SBP<130, DBP<85  SBP:130-139 or DBP:85-89  SBP:140-159 or DBP:90-99  SBP≥160 or DBP≥100   - Yes   SBP<130, DBP<85  SBP:130-139 or DBP:85-89  SBP:140-159 or DBP:90-99  SBP≥160 or DBP≥100 | Ref  0.120  0.460  0.720  0.990  1.060  1.330  1.650 | -  -  -  -  -  -  -  - | Ref  0.087  0.336  0.527  0.725  0.776  0.974  1.208 | Ref  0.087  0.336  0.527  0.725  0.776  0.974  1.208 | Ref  0.087  0.336  0.527  0.725  0.776  0.974  1.208 | Ref  -0.179  0.200  0.097  -0.647  0.011  -0.100  0.239 | -  -  -  -  -  -  -  - |
|  | Hyperlipidemia medication   - No   TC: <200 mg/dL  TC: 200-239 mg/dL  TC: > 240 mg/dL   - Yes   TC: <200 mg/dL  TC: 200-239 mg/dL  TC: > 240 mg/dL | Ref  0.340  0.530  0.360  0.350  0.910 | -  -  -  -  -  - | Ref  0.249  0.388  0.264  0.256  0.666 | Ref  0.249  0.388  0.264  0.256  0.666 | Ref  0.249  0.388  0.264  0.256  0.666 | Ref  -0.062  -0.548  -0.124  0.315  -0.327 | Ref  -  -0.561  -  -  - |
|  | Variation in HbA1c, %   - < 8.5 - 8.5 – 17.5 - > 17.5 | Ref  -  - | -  -  - | -  -  - | -  -  - | -  -  - | -  -  - | -  -  - |
|  | Variation in SBP, %   - < 4.4 - 4.4 – 8.7 - > 8.7 | Ref  -  - | -  -  - | -  -  - | -  -  - | -  -  - | -  -  - | -  -  - |
|  | BMI, kg/m^2^  FPG, mg/dL | -  - | -  - | -  - | -  - | -0.054  0.003 | -  - | -  - |
| Wan, Chinese ESRD Risk Score  (Male Equations) | Intercept | - | - | -3.921 | -3.921 | -3.921 | -1.745 | -1.549 |
|  | Age, years | 0.060 | - | 0.027 | -0.041 | 0.027 | -0.006 | - |
|  | Smoking   - Non-Smoker - Smoker | Ref  0.258 | -  - | Ref  0.117 | Ref  0.117 | Ref  0.117 | Ref  -0.113 | -  - |
|  | Retinopathy   - No - Yes | Ref  0.386 | -  - | Ref  0.176 | Ref  0.176 | Ref  0.176 | Ref  0.698 | Ref  0.699 |
|  | Anti-HT drugs   - No - Yes | Ref  0.433 | -  - | Ref  0.197 | Ref  0.197 | Ref  0.197 | Ref  -0.009 | -  - |
|  | Insulin used   - No - Yes | Ref  2.815 | -  - | Ref  1.286 | Ref  2.152 | Ref  1.939 | Ref  1.641 | Ref  0.797 |
|  | Oral diabetic drugs   - No - Yes | Ref  0.315 | -  - | Ref  0.144 | Ref  0.144 | Ref  0.144 | Ref  0.169 | -  - |
|  | SBP, mmHg | 0.010 | - | 0.004 | 0.004 | 0.004 | -0.008 | - |
|  | DBP, mmHg | -0.074 | - | -0.033 | -0.033 | -0.033 | 0.258 | - |
|  | DBP^2^, mmHg | 0.0001 | - | 0.00004 | 0.00004 | 0.00004 | -0.0006 | - |
|  | eGFR, mL/min/1.73 m^2^   - ≥ 90 - 60-89 - < 60 | Ref  2.169  0.989 | -  -  - | Ref  0.991  0.452 | Ref  0.613  -1.894 | Ref  -0.286  -1.575 | Ref  -1.451  -2.304 | Ref  -1.341  -2.020 |
|  | Age*Insulin used | -0.029 | - | -0.013 | -0.013 | -0.013 | -0.014 | - |
|  | HbA1c, % | -0.235 | - | - | - | - | - | - |
|  | HbA1c^2^, % | 0.019 | - | - | - | - | - | - |
|  | Ln (UACR+1), mg/mmol | 1.147 | - | - | - | - | - | - |
|  | LDL, mg/dL  FPG, mg/dL  Albuminuria   - Normo-albuminuria - Microalbuminuria - Macroalbuminuria - Adv. Albuminuria | -  -  -  -  -  - | -  -  -  -  -  - | -  -  -  -  -  - | -  -  -  -  -  - | -0.089  0.004  Ref.  0.544  0.788  1.276 | -  -  -  -  -  - | -  -  -  -  -  - |
| Wan, Chinese ESRD Risk Score  (Female Equations) | Intercept | - | - | -2.410 | -2.410 | -2.410 | -2.945 | -1.855 |
|  | Age, years | 0.034 | - | 0.022 | -0.021 | 0.022 | -0.023 | - |
|  | Diabetic durations, years | 0.011 | - | 0.007 | 0.007 | 0.007 | -0.027 | - |
|  | Anti-HT drugs   - No - Yes | Ref  0.503 | -  - | Ref  0.327 | Ref  -0.421 | Ref  0.327 | Ref  -0.198 | -  - |
|  | Insulin used   - No - Yes | Ref  0.545 | -  - | Ref  0.355 | Ref  1.686 | Ref  0.355 | Ref  1.658 | Ref  1.713 |
|  | Oral diabetic drugs   - No - Yes | Ref  0.684 | -  - | Ref  0.445 | Ref  0.445 | Ref  0.445 | Ref  0.665 | -  - |
|  | BMI, kg/m^2^ | -0.157 | - | -0.102 | -0.102 | -0.102 | -0.016 | - |
|  | BMI^2^, kg/m^2^ | 0.003 | - | 0.001 | 0.001 | 0.001 | -0.0007 | -0.0009 |
|  | SBP, mmHg | 0.005 | - | 0.003 | 0.003 | 0.003 | 0.019 | - |
|  | DBP, mmHg | -0.086 | - | 0.056 | 0.056 | 0.056 | -0.018 | - |
|  | DBP^2^, mmHg | 0.0005 | - | 0.0003 | 0.0003 | 0.0003 | 0.0001 | - |
|  | eGFR, mL/min/1.73 m^2^   - ≥ 90 - 60-89 - < 60 | Ref  0.693  4.719 | -  -  - | Ref  0.451  3.076 | Ref  -0.609  2.091 | Ref  0.451  3.076 | Ref  -0.818  3.804 | Ref  -  1.987 |
|  | Age interaction term:  Age* eGFR (60-89)  Age* eGFR (<60) | 0.002  -0.031 | -  - | 0.001  -0.020 | 0.001  -0.020 | 0.001  -0.020 | -0.004  -0.020 | -  - |
|  | HbA1c, % | -0.356 | - | - | - | - | - | - |
|  | HbA1c^2^, % | 0.029 | - | - | - | - | - | - |
|  | Ln (UACR+1), mg/mmol | 0.371 | - | - | - | - | - | - |
|  | FPG, mg/dL |  |  |  |  | 0.003 |  |  |
| Elley, New Zealand DCS Risk Score | Intercept | -6.695 | -8.922 | -8.922 | -8.922 | -8.922 | -4.152 | -5.013 |
|  | Age DM onset, years | 0.024 | 0.024 | 0.032 | 0.007 | 0.032 | -0.007 | - |
|  | Sex   - Female - Male | 0.401  Ref | 0.401  Ref | 0.541  Ref | 0.541  Ref | 0.541  Ref | 0.136  Ref | -  - |
|  | Ethnicity   - East Asian - Others’ ethnicity | -0.138  - | -0.138  - | -0.186  - | -0.186  - | -0.186  - | -0.948  - | -  - |
|  | Diabetic duration, years | 0.043 | 0.043 | 0.058 | 0.058 | 0.058 | 0.300 | 0.032 |
|  | Serum Creatinine, µmol/L | 0.305 | 0.305 | 0.411 | 0.411 | 0.601 | 0.202 | 0.197 |
|  | Albuminuria   - Normo-albuminuria - Microalbuminuria - Macroalbuminuria - Adv. Albuminuria | Ref  0.644  1.303  2.290 | Ref  0.644  1.303  2.290 | Ref  0.869  1.758  3.090 | Ref  0.869  1.758  3.090 | Ref  1.393  2.541  3.090 | Ref  0.648  0.822  - | Ref  0.636  -  - |
|  | SBP, mmHg | 0.049 | 0.049 | 0.066 | 0.066 | 0.066 | 0.029 | - |
|  | Smoking   - Nonsmoker - Ex Smoking - Current smoking | Ref  -0.003  0.252 | Ref  -0.003  0.252 | Ref  -0.004  0.340 | Ref  -0.004  0.340 | Ref  -0.004  0.340 | Ref  -0.095  -0.151 | -  -  - |
|  | Previous CVD history   - No - Yes | Ref  0.498 | Ref  0.498 | Ref  0.672 | Ref  0.672 | Ref  0.672 | Ref  0.118 | -  - |
|  | HbA1c, % | 0.173 | 0.173 | - | - | - | - | - |
|  | BMI, kg/m^2^  Oral DM drug  FPG, mg/dL | -  -  - | -  -  - | -  -  - | -  -  - | 0.057  0.590  0.003 | -  -  - | -  -  - |

Abbreviations: BMI, body mass index; CVD, cardiovascular disease; DBP, diastolic blood pressure; DLP, dyslipidemia; DM, diabetic mellitus; eGFR, estimated glomerular filtration rate; FPG, fasting plasma glucose; HbA1c, glycated hemoglobin; HDL-C, high density lipoprotein- cholesterol; HT, hypertension; LDL, low density lipoprotein; SBP, systolic blood pressure; TC, Total Cholesterol; UACR, urine albumin to creatinine ratio

### **Table S8.** Prognostic model discrimination performance comparisons between baseline validation (M_0_) and updated models (M_4_)

| **Study** | **Prognostic Model** | **Prognostic factors** | **C-statistics**  **(95% CI)** | **∆ C-statistic**  **(95% CI)** |
| --- | --- | --- | --- | --- |
|  |  |  |  |  |
| **Chronic Kidney Disease (CKD)** | | | | |
| Wu, et al. | Established (M_0_) | sex, BMI, SBP, duration | 0.585 (0.565, 0.605) | Ref. |
|  | Update (M_4_) | sex, BMI, SBP, duration, FPG group*Oral DM drug | 0.790 (0.774, 0.806) | 0.214 (0.193, 0.234) |
| Miao, et al.  (Male Equation) | Established (M_0_) | age, BMI, Cr, HDL, location, DR, physical activity, DLP | 0.720 (0.691, 0.749) | Ref. |
|  | Update (M_4_) | age, BMI, Cr, HDL, location, DR, physical activity, DLP, FPG group, Oral DM drug | 0.796 (0.769, 0.822) | 0.072 (0.050, 0.947) |
| Miao, et al.  (Female Equation) | Established (M_0_) | age, Cr, HDL, Location, DLP, DR | 0.786 (0.765, 0.806) | Ref. |
|  | Update (M_4_) | age, Cr, HDL, Location, DLP, DR, FPG group*Oral DM drug | 0.831 (0.812, 0.851) | 0.041 (0.027, 0.547) |
| Low, et al. | Established (M_0_) | age, SBP, e-GFR, LDL | 0.707 (0.689, 0.726) | Ref. |
|  | Update (M_4_) | age, SBP, e-GFR, LDL, FPG group*Oral DM drug | 0.822 (0.806, 0.837) | 0.114 (0.102, 0.126) |
| **End Stage Renal Disease (ESRD)** | | | | |
| Lin, et al. | Established (M_0_) | age on set, sex, Cr, SBP, DR, albuminuria, insulin, oral DM drug, anti-HT drug | 0.671 (0.626, 0.717) | Ref. |
|  | Update (M_4_) | age on set, sex, Cr, SBP, DR, albuminuria, insulin, oral DM drug, anti-HT drug, FPG, BMI | 0.759 (0.716, 0.801) | 0.089 (0.062, 0.116) |
| Wan, et al.  (Male Equation) | Established (M_0_) | age, smoking, DR, HT drug, Oral DM drug, Insulin, SBP, DBP, eGFR | 0.700 (0.639, 0.761) | Ref. |
|  | Update (M_4_) | age, smoking, DR, HT drug, Oral DM drug, Insulin, SBP, DBP, eGFR, FPG, LDL | 0.774 (0.717, 0.830) | 0.064 (0.035, 0.093) |
| Wan, et al. (Female Equation) | Established (M_0_) | age, smoking, DR, HT drug, Oral DM drug, Insulin, SBP, DBP, eGFR, duration, BMI | 0.760 (0.705, 0.816) | Ref. |
|  | Update (M_4_) | age, smoking, DR, HT drug, Oral DM drug, Insulin, SBP, DBP, eGFR, duration, BMI, FPG | 0.806 (0.755, 0.857) | 0.039 (0.016, 0.062) |
| Elley, et al. | Established (M_0_) | sex, ethnicity, age, duration, Cr, albuminuria, SBP, smoking, CVD | 0.744 (0.701, 0.788) | Ref. |
|  | Update (M_4_) | sex, ethnicity, age, duration, Cr, SBP, smoking, albuminuria, CVD, BMI, FPG, Oral DM drug | 0.774 (0.734, 0.814) | 0.025 (0.016, 0.035) |

Note: C-statistics based on 1,000 iterations; ∆ C-statistic, C-statistic change comparing to established model; *) interaction effects

Abbreviations: BMI, body mass index; Cr, creatinine; CVD, Cardiovascular diseases; DBP, diastolic blood pressure; DR, diabetic retinopathy; DLP, dyslipidemia; DM, diabetic mellitus; eGFR, estimated glomerular filtration rate; FPG, fasting plasma glucose; HDL, high density lipoprotein; HT, hypertension; LDL, low density lipoprotein; SBP, systolic blood pressure

## Supplementary Figures

### **Figure S1.** PRISMA flowchart for the identification and selection of CKD prognostic models

### **Figure S2.** Stepwise Receiving Operating Characteristic (ROC) Curve for each prognostic model validation (M_0_ – M_6_)


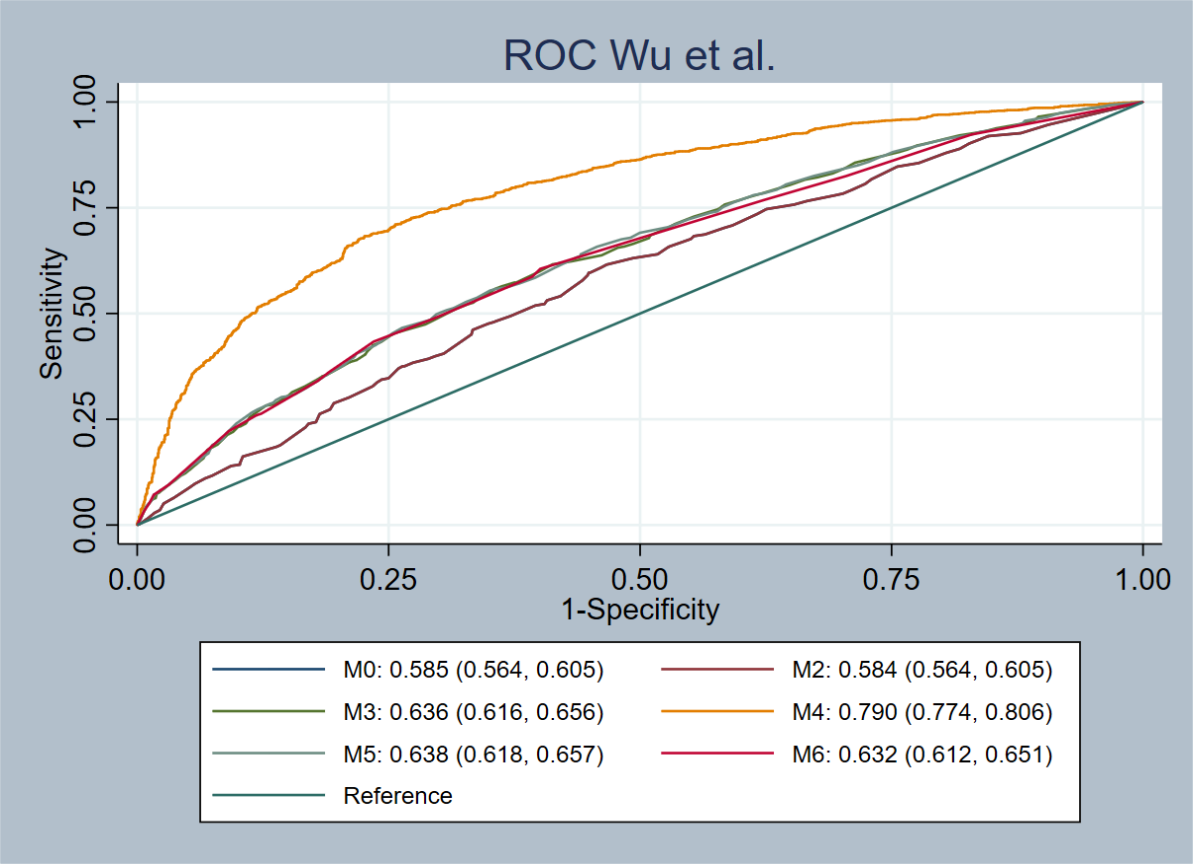


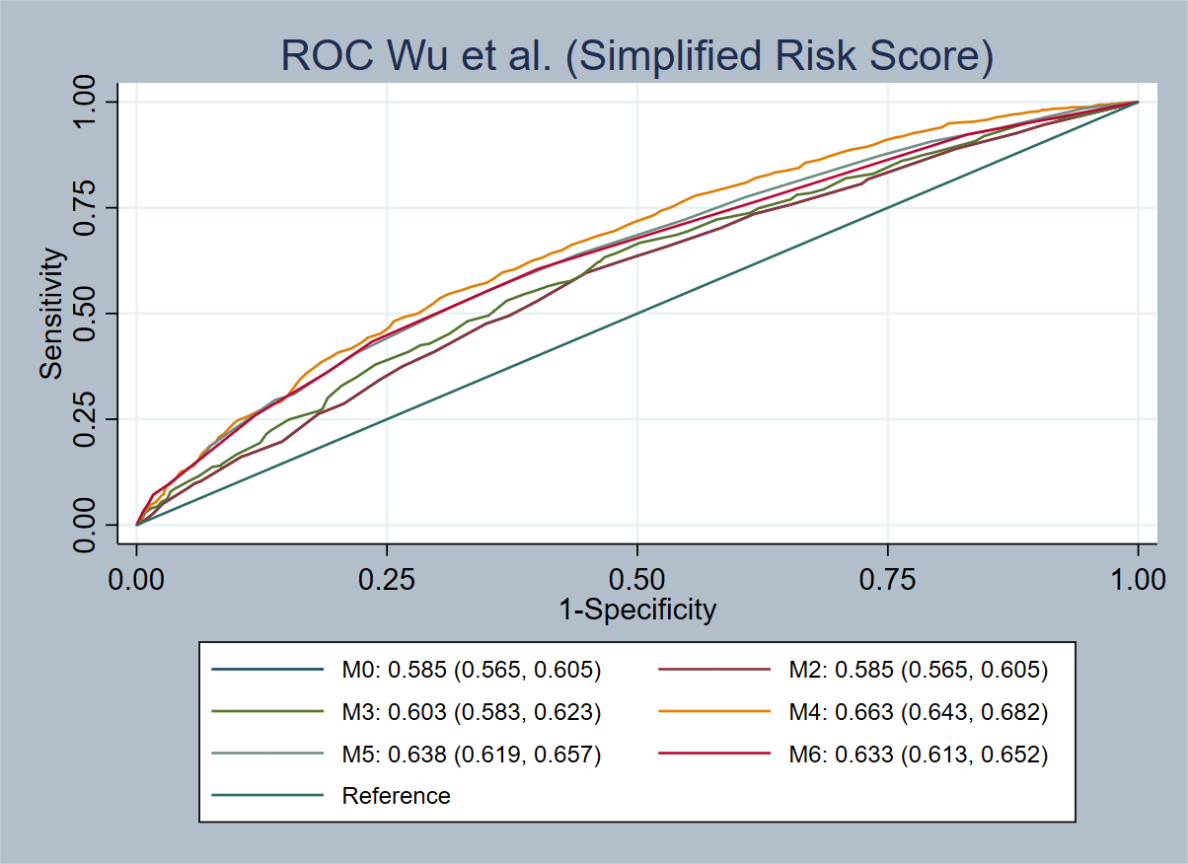


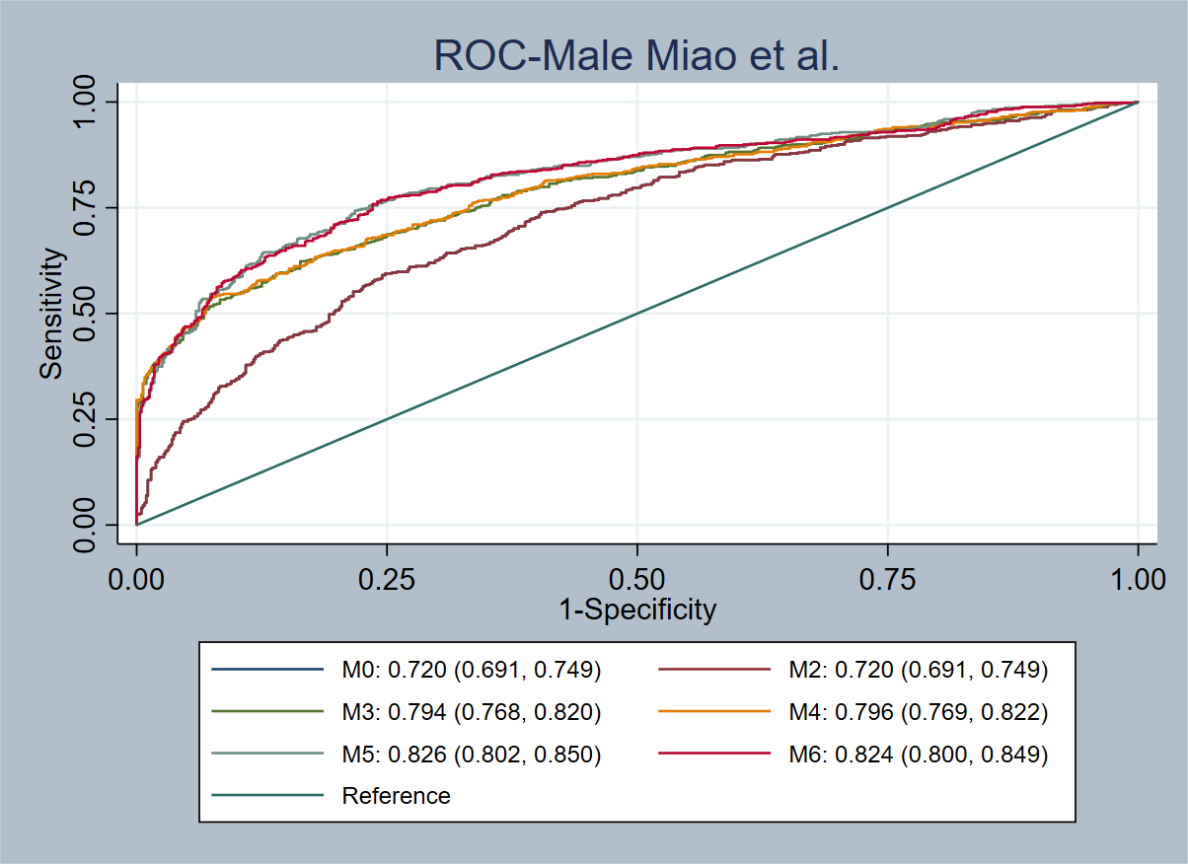


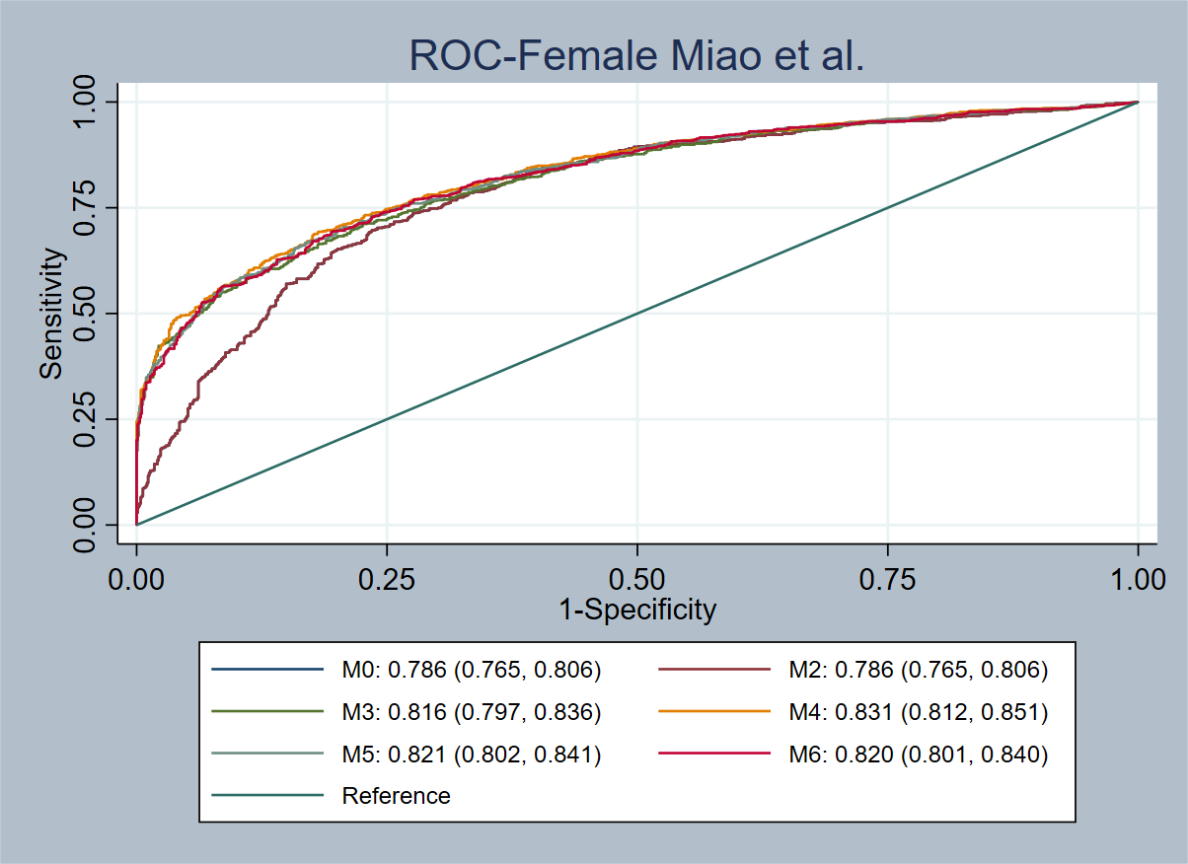


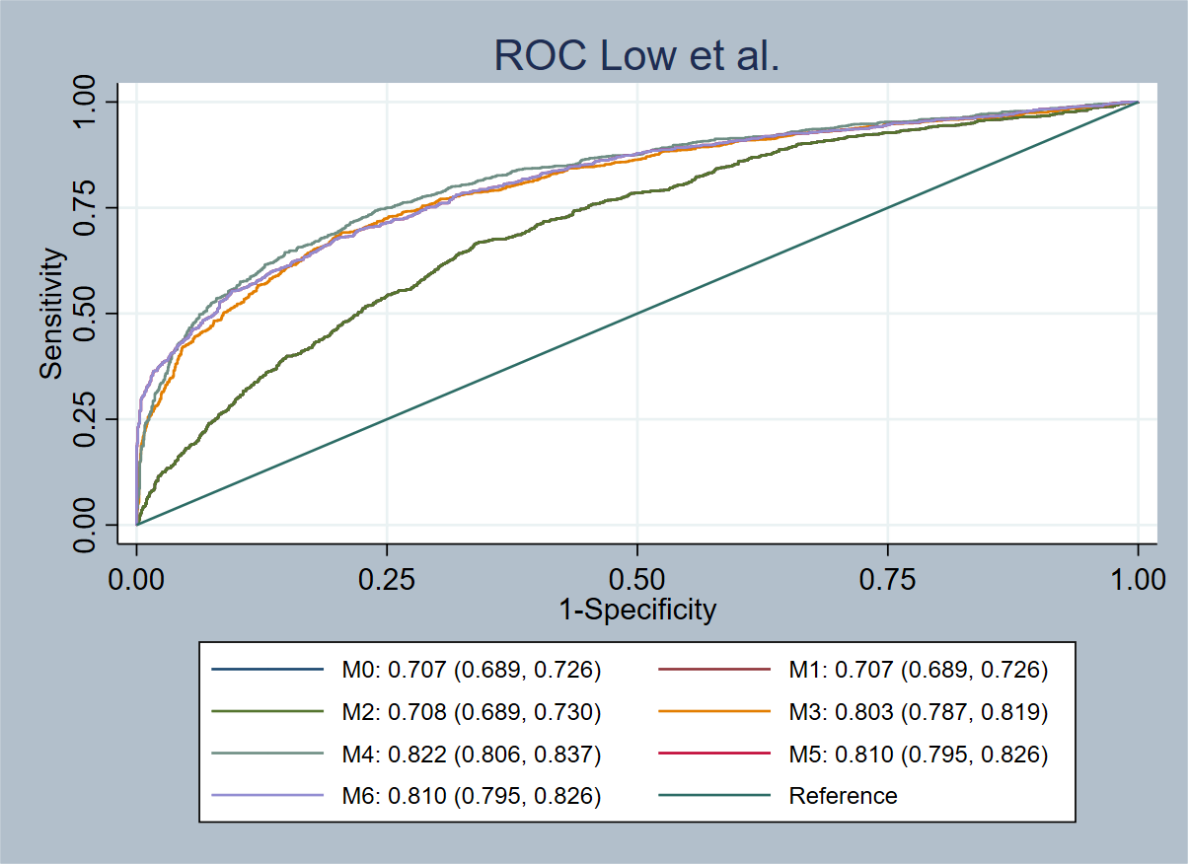


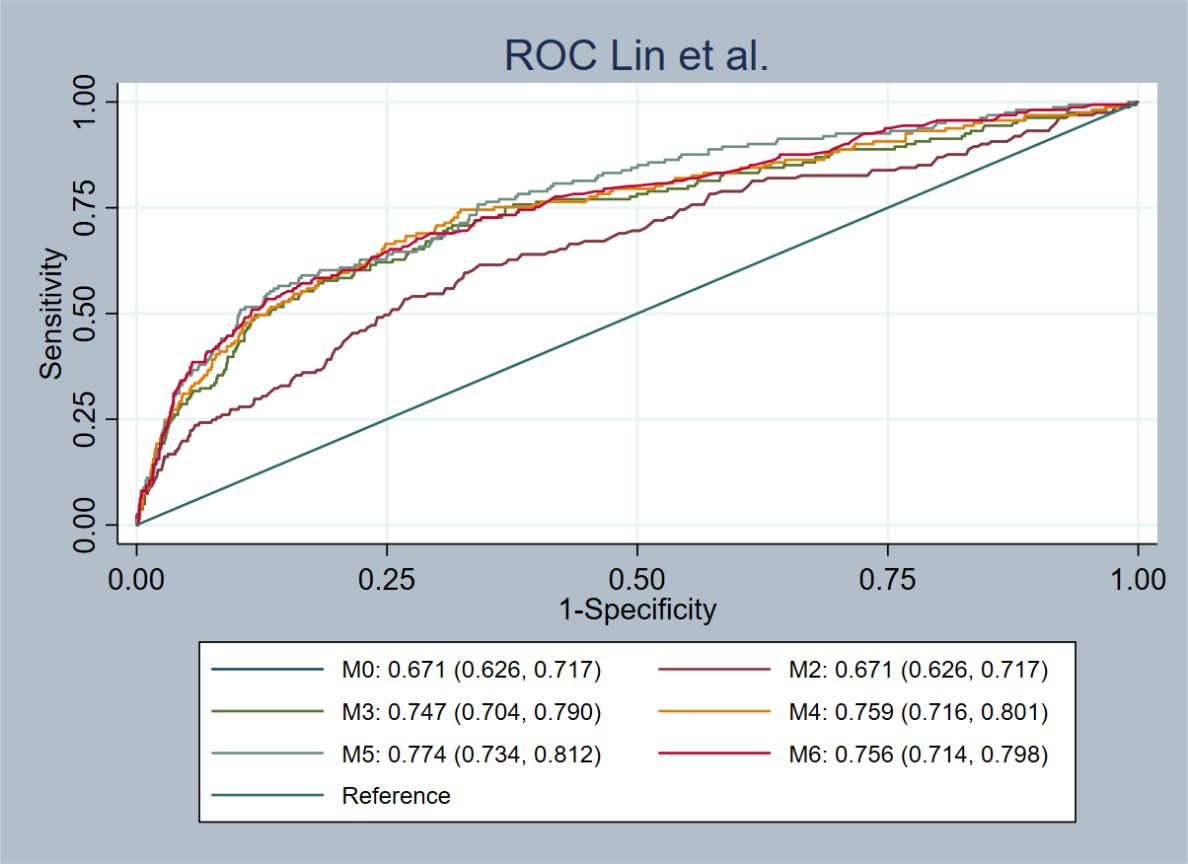


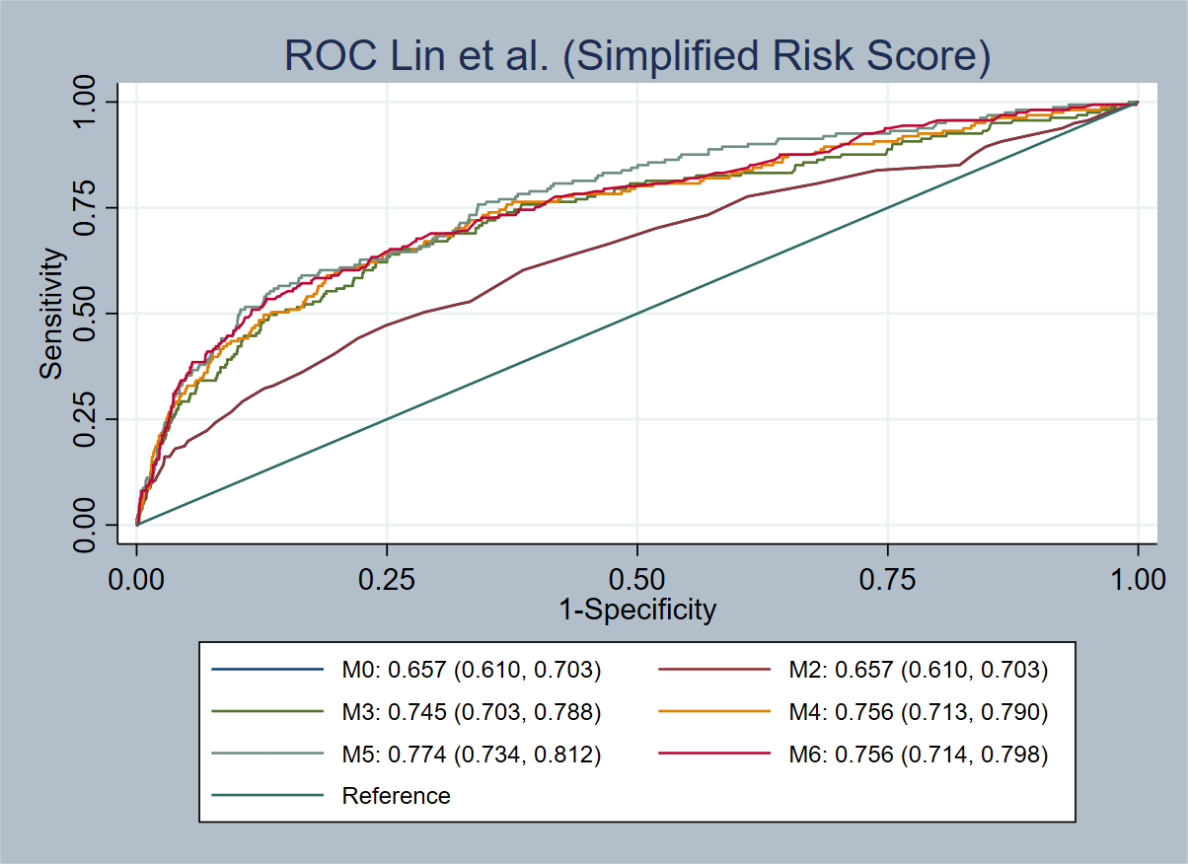


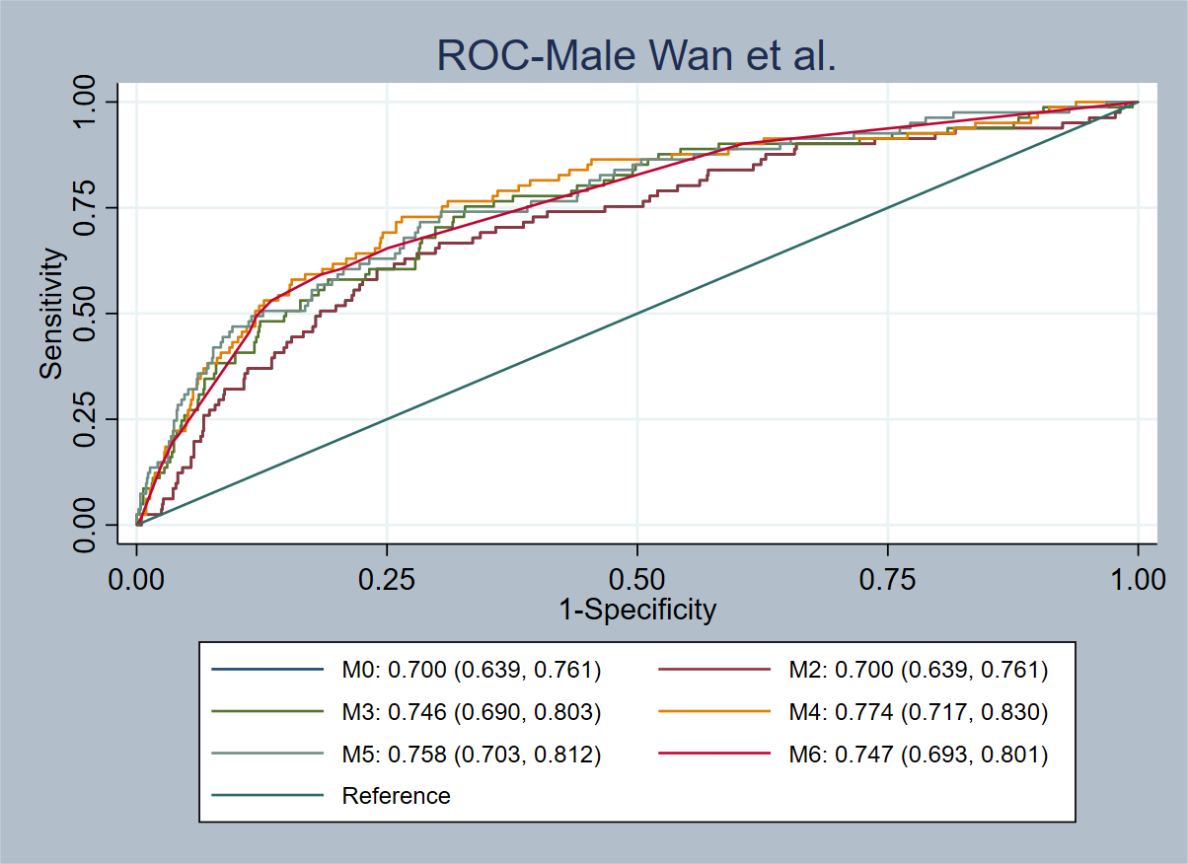


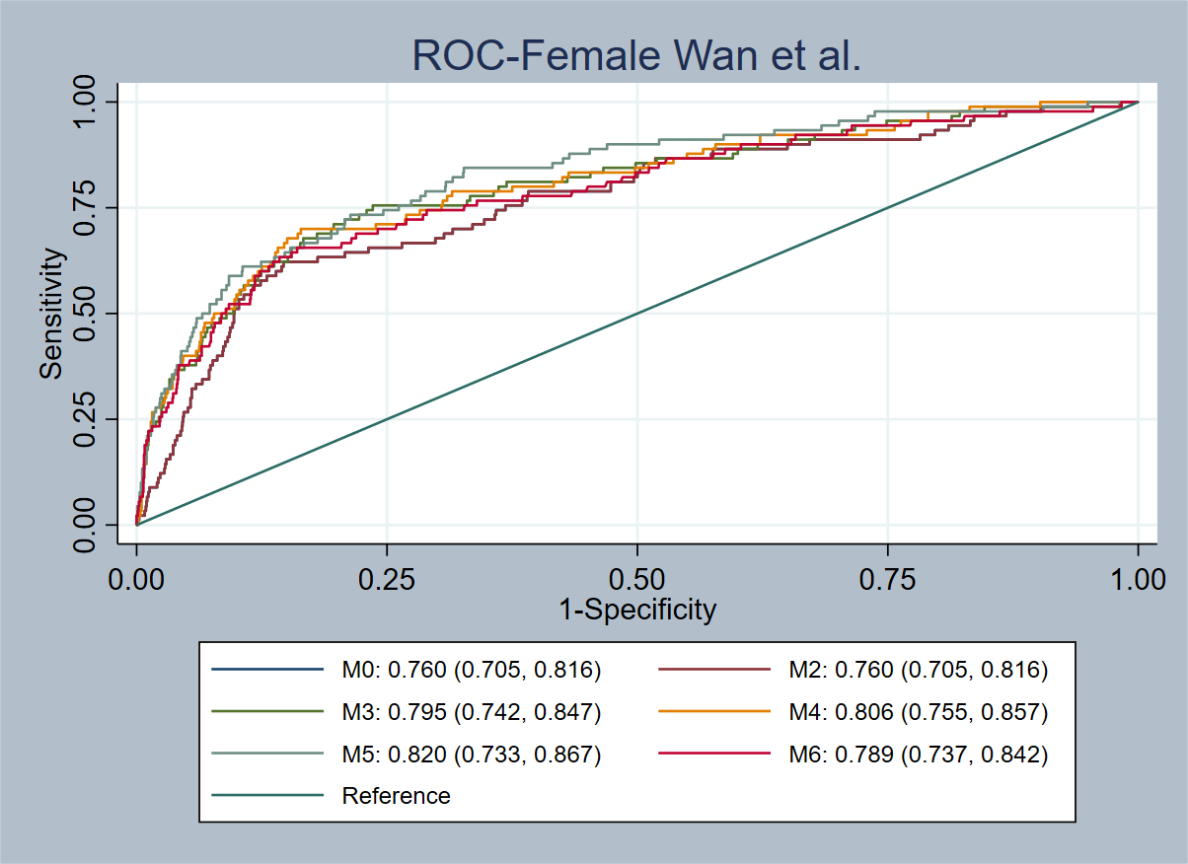


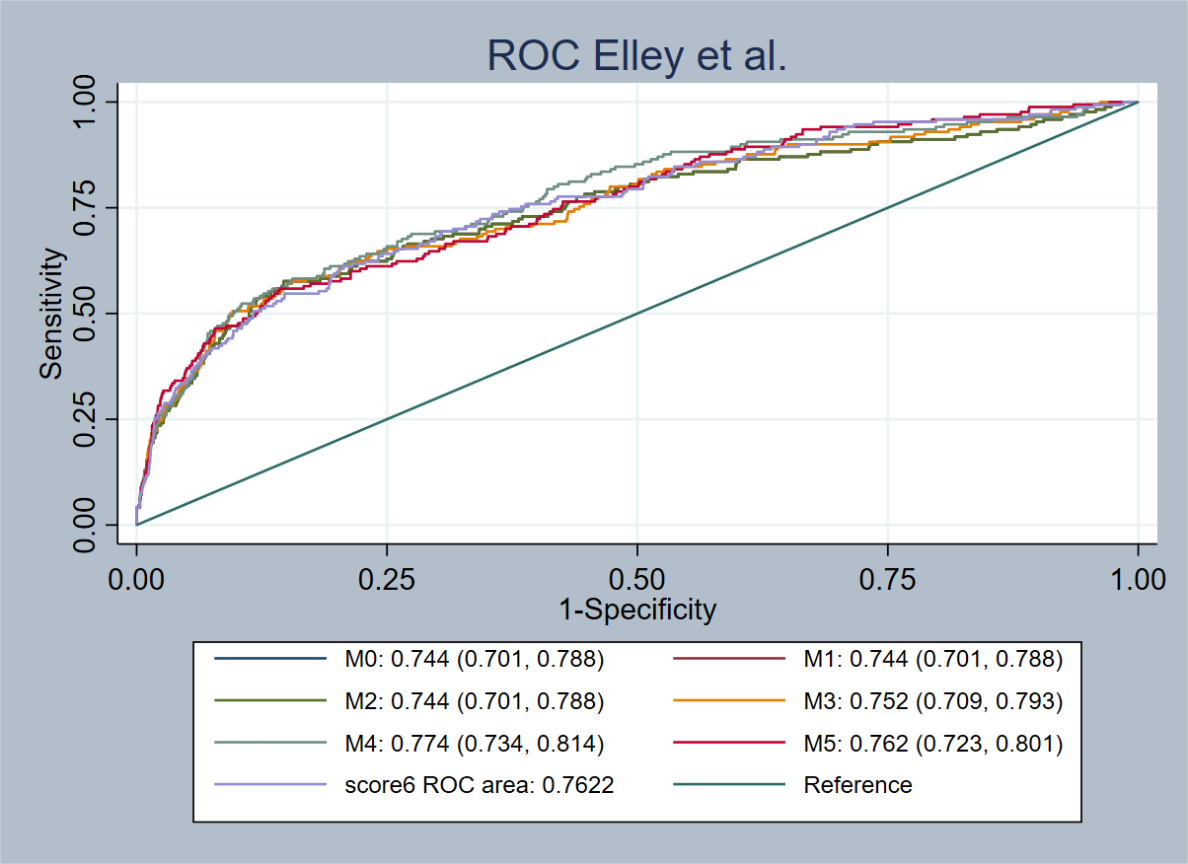


### **Figure S3.** Stepwise calibration plots for each prognostic model validation (M_0_ – M_6_)


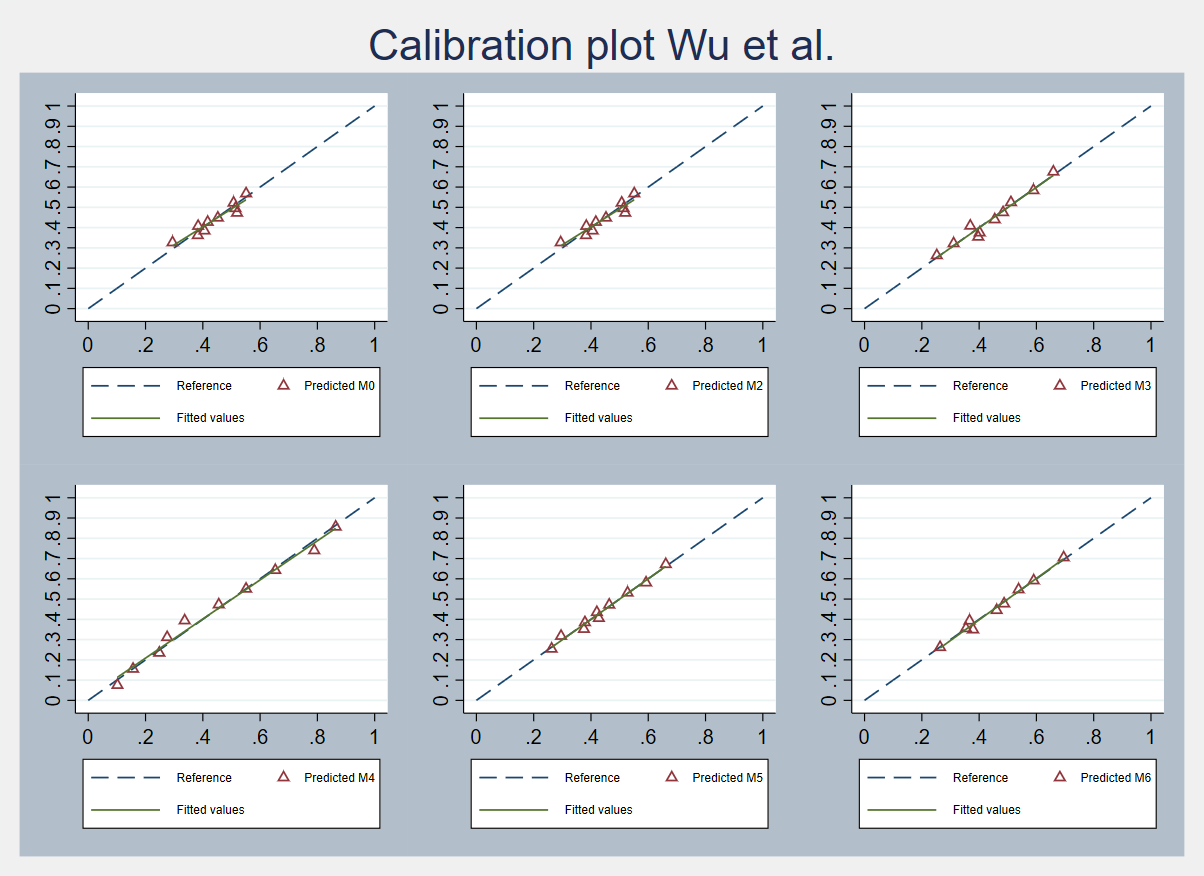


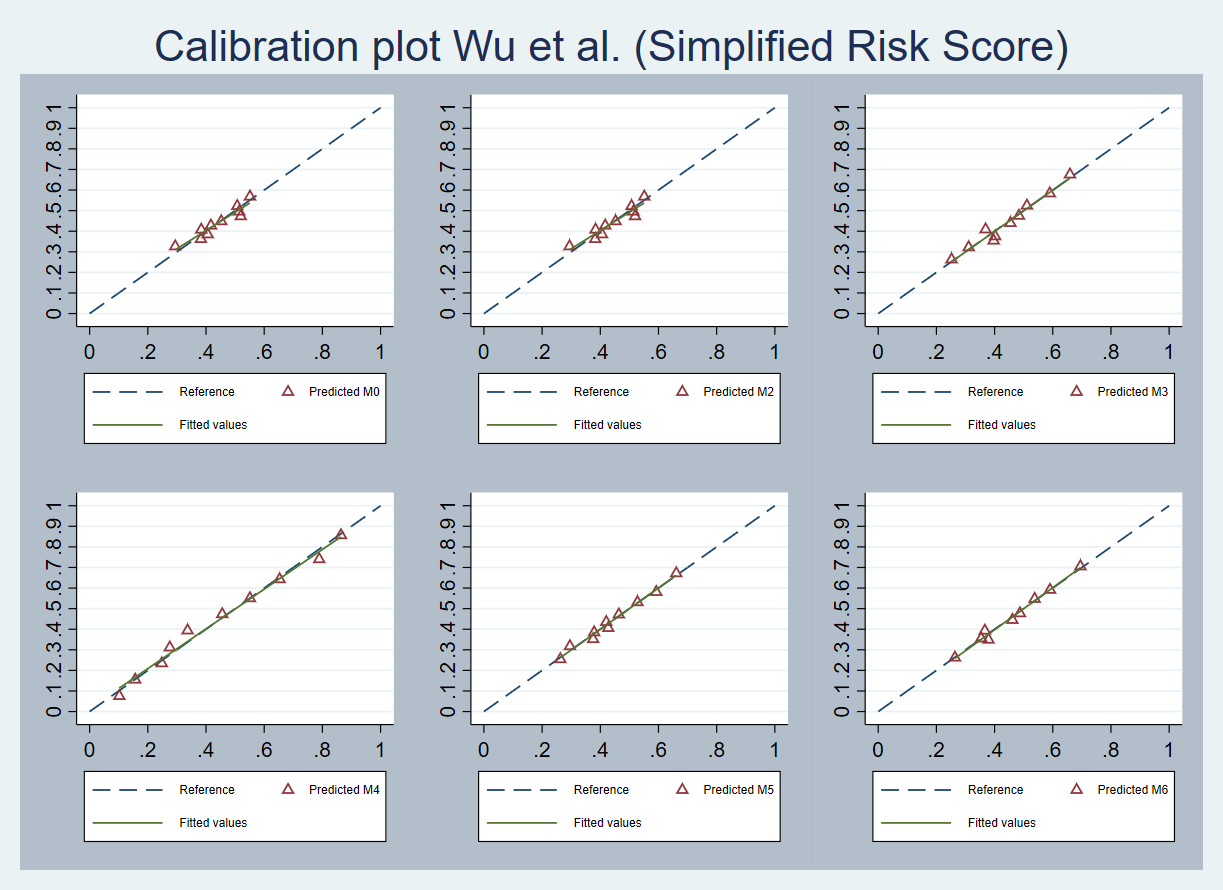


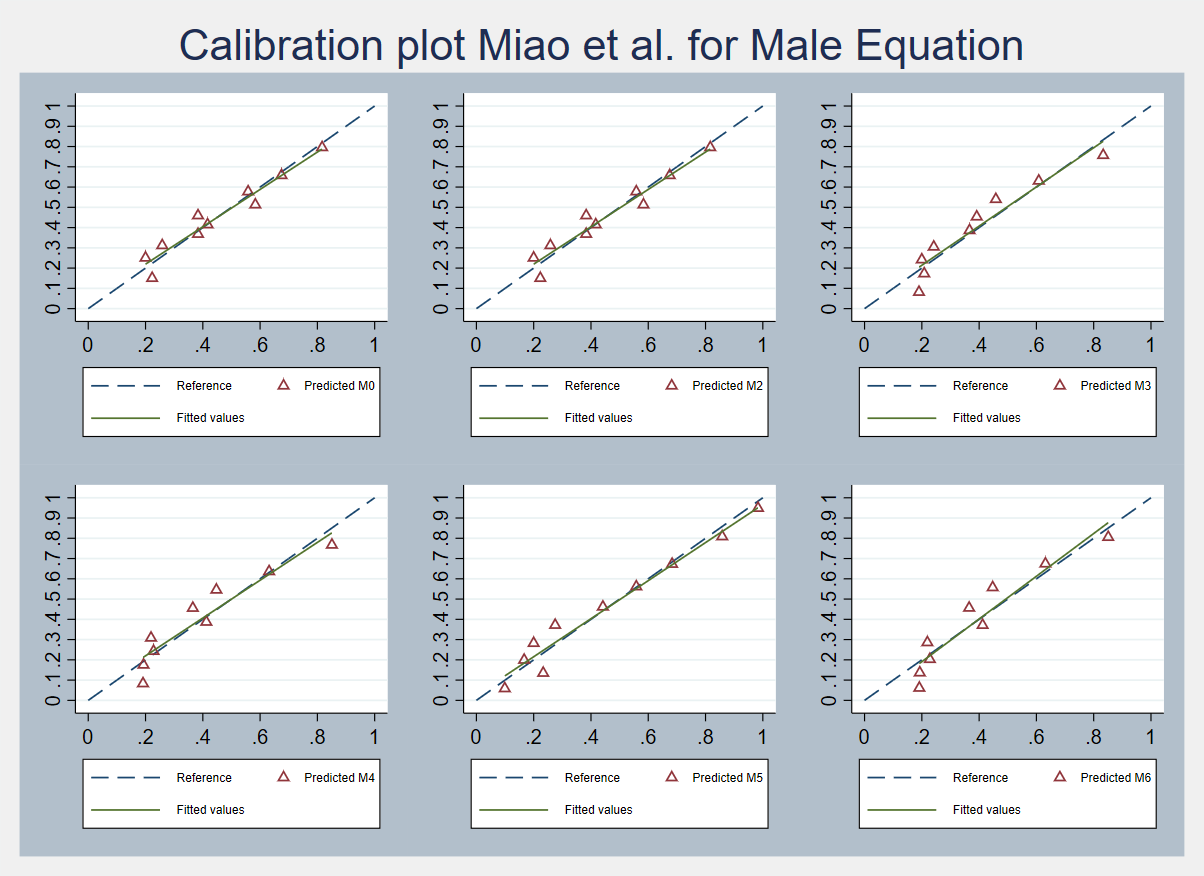


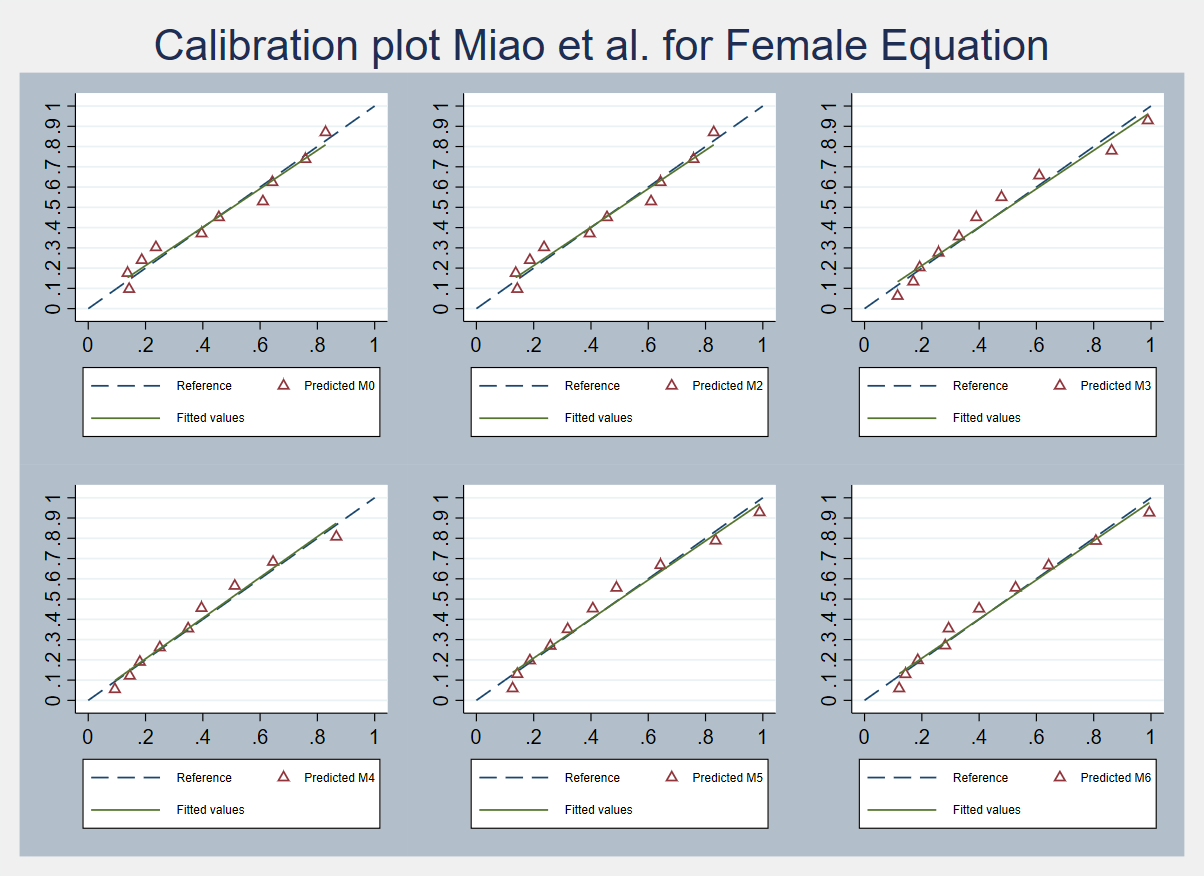


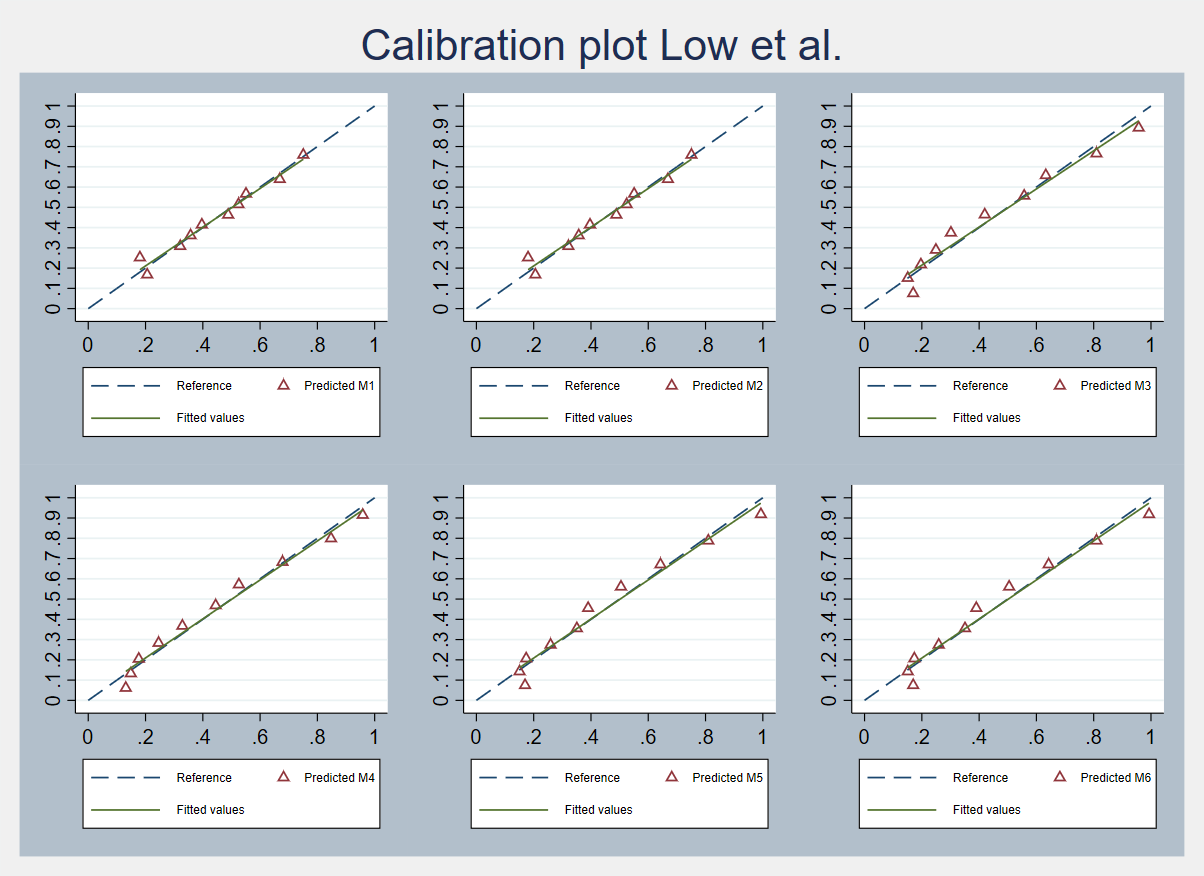


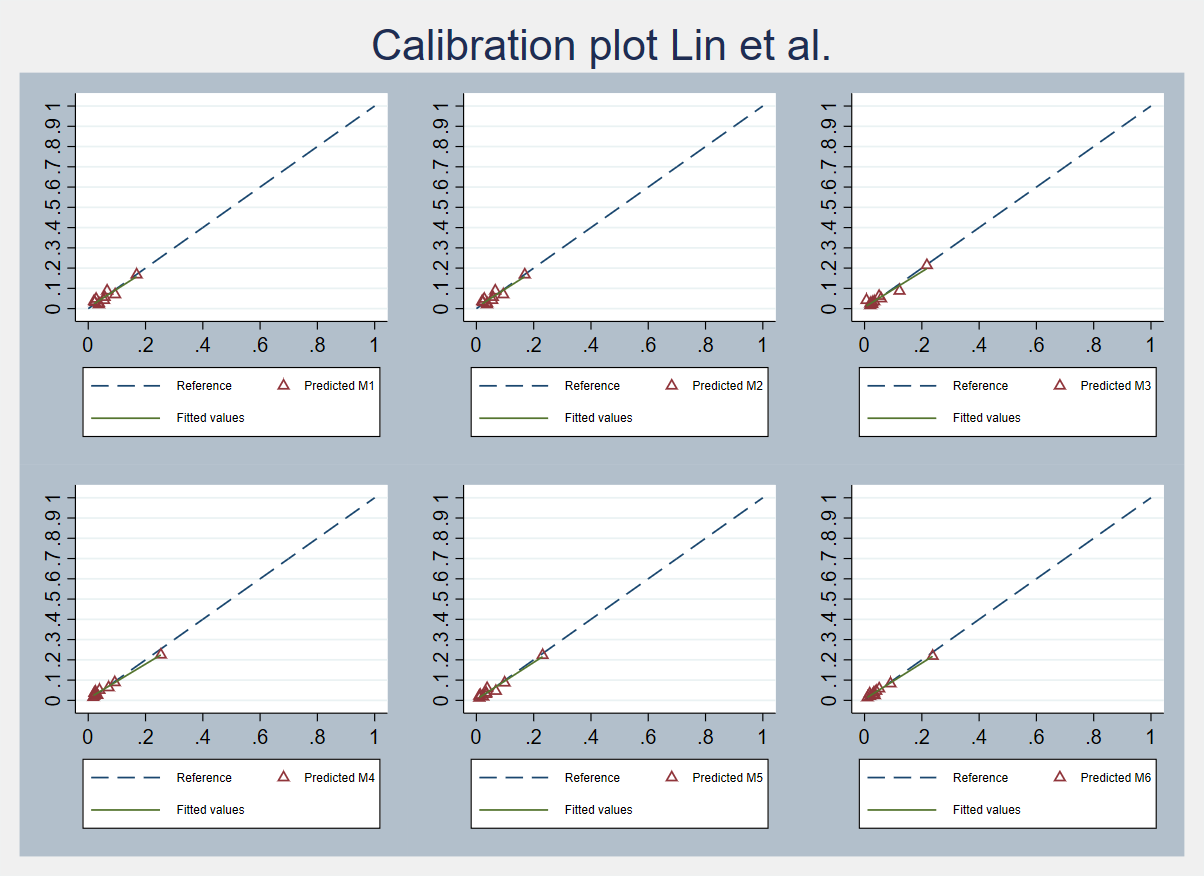


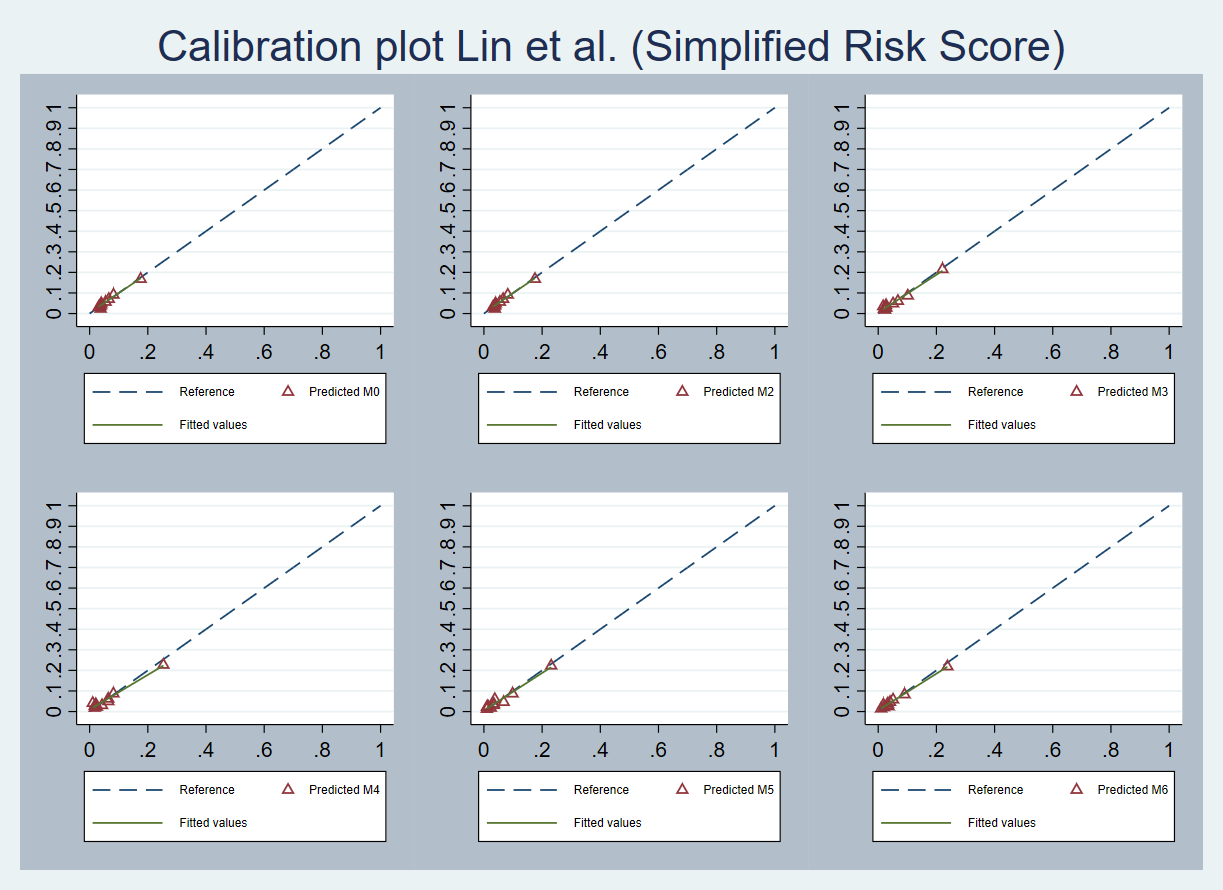


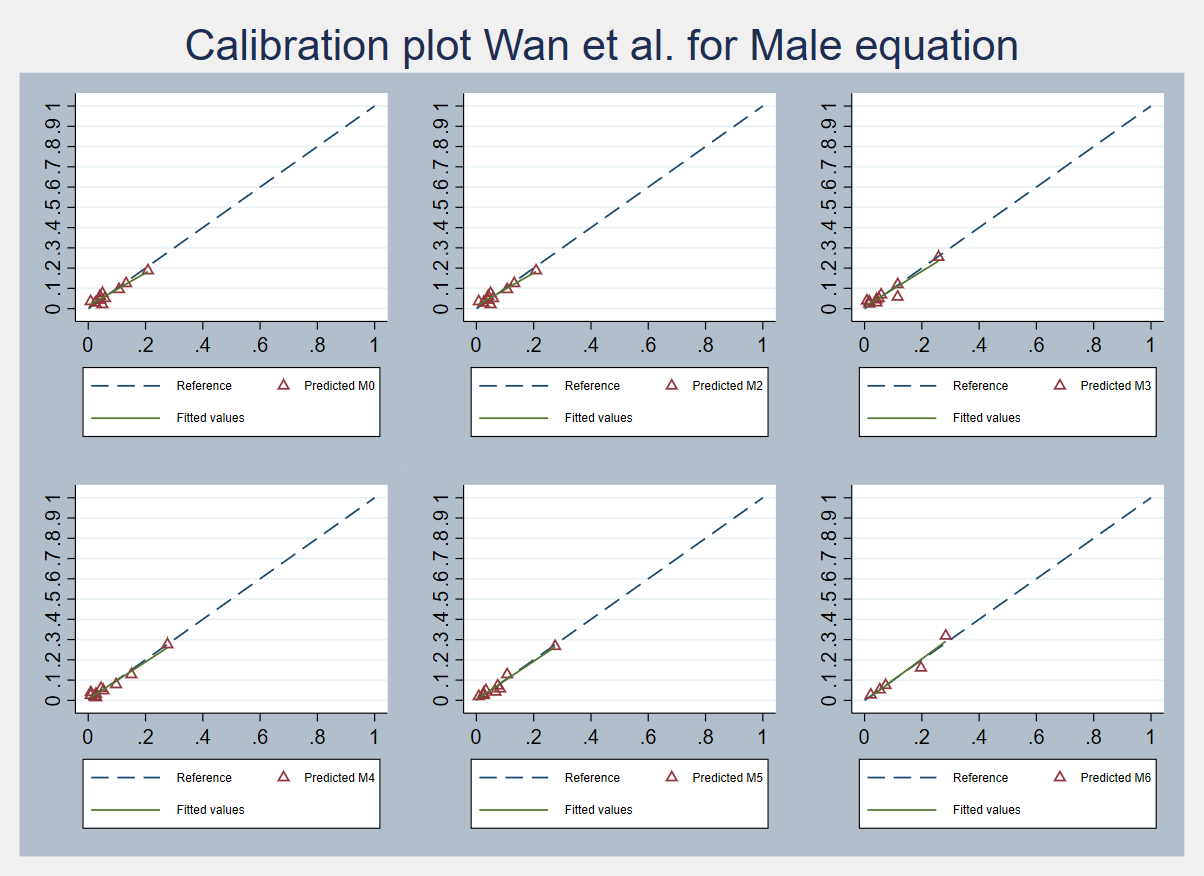


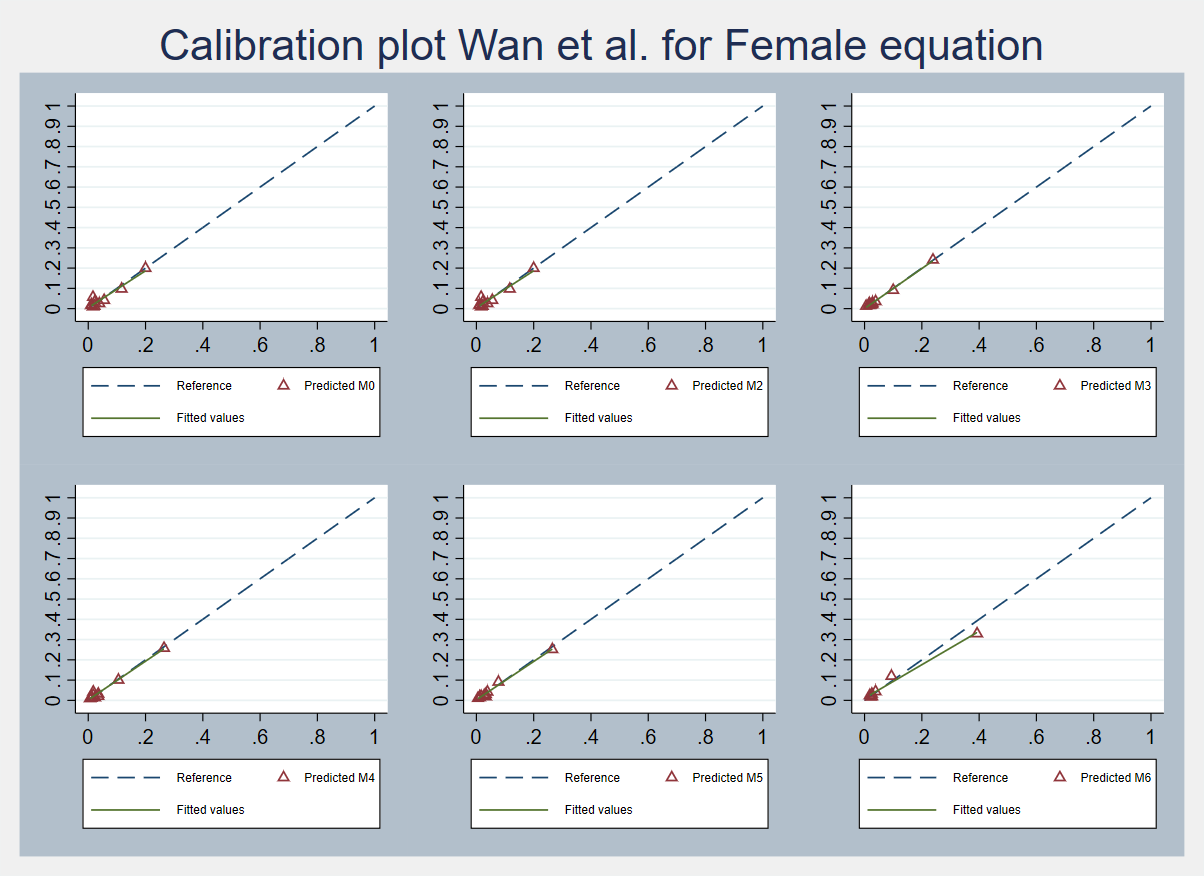


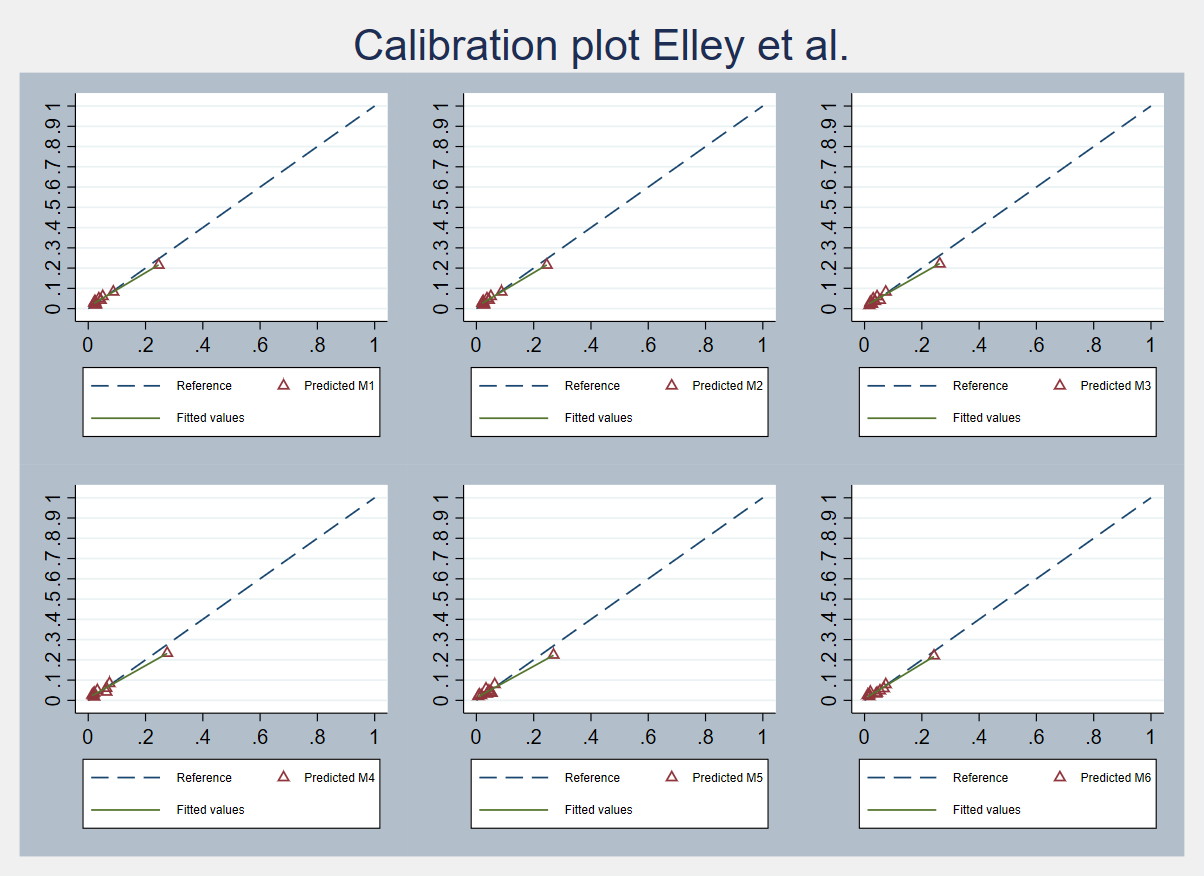

Supplement: Supplementary file 1 — Supplementary file1 (DOCX 34215 KB) [file 40620_2021_1220_MOESM1_ESM.docx]
